# CCKergic Tufted Cells Regulate Odor Sensitivity by Controlling Mitral Cell Output in the Mouse Olfactory Bulb

**DOI:** 10.1523/JNEUROSCI.1243-24.2025

**Published:** 2025-03-24

**Authors:** Eric Starr, Rashika Budhathoki, Dylan Gilhooly, Laura Castillo, Meigeng Hu, Dan Zhao, Yaping Li, Shaolin Liu

**Affiliations:** ^1^Department of Anatomy, Howard University, Washington, DC 20059; ^2^Center for Neurological Disease Research, Departments of Physiology and Pharmacology and Biomedical Sciences, University of Georgia College of Veterinary Medicine, Athens, Georgia 30602

**Keywords:** gap junction, mitral cell, neural circuits, odor detection, olfactory bulb, synaptic transmission

## Abstract

Despite the importance of odor detection to the survival of most animals, mechanisms governing olfactory sensitivity remain unclear, especially beyond the olfactory sensory neurons. Here, we leverage opto- and chemo-genetics to selectively modulate activities of CCKergic tufted cells (TCs) in the mouse olfactory bulb (OB) of either sex, which form the intrabulbar associational system (IAS) to link isofunctional glomeruli, to determine the functional impact on OB output via mitral cells (MCs) and odor detection in behaving animals. NMDA receptors in CCKergic TCs remarkably amplify the OSN-evoked monosynaptic responses in these excitatory neurons, which provide a long-lasting feedforward excitation to MCs via both chemical transmission and electrical synapses between their apical dendrites. NMDA receptors in MCs mediate late components of the dendrodendritic TC→MC transmission to significantly boost MC output. Congruently, optogenetic inhibition of the CCKerigic TCs dramatically reduces the OSN-evoked MC responses. Unexpectedly, optogenetic activation of the axons projecting from CCKergic TCs on the opposite side of the same bulb produces mainly AMPA receptor-mediated excitatory responses in MCs, leading us to speculate that CCKergic TCs functionally synchronize MC output from mirror glomeruli. Furthermore, chemogenetic inhibition of CCKergic TCs reduces animal’s sensitivity to odors by elevating the detection threshold, consistent with the key role of these TCs in functionally controlling MC output. Collectively, our results delineate the cellular and circuit mechanisms allowing the CCKergic TCs to regulate MC output from glomeruli on both the medial and lateral sides of each OB and the system’s sensitivity to odors possibly via the IAS.

## Significance Statement

The detection and processing of chemical stimuli, such as environmental odorants, are essential for the central nervous system to generate appropriate behavioral responses in animals. Most of our current knowledge about odor detection comes from studies on the interactions between chemical stimuli and odorant receptors (ORs) on olfactory sensory neurons (OSNs) at the periphery. In this study, we have identified a specific subpopulation of nerve cells that play a crucial role in converting sensory input into biological signals within the olfactory bulb, the downstream target of OSNs, and the initial site of synaptic odor processing. Our findings provide new insights into the cellular and circuit-level mechanisms that regulate olfactory detection beyond sensory neurons.

## Introduction

Given the importance of detecting chemical stimuli in the environment to the survival of most animals (Lledo et al., 2005), understanding how the olfactory system governs its sensitivity to odorants is of paramount significance. Previous studies on this topic mainly focused on odorant receptors (ORs) on olfactory sensory neurons (OSNs) in the olfactory epithelium (van Drongelen et al., 1978; Meisami, 1989; Apfelbach et al., 1991; Dekker et al., 2006; Dewan et al., 2018). However, the neurobiological mechanisms underlying olfactory sensitivity and odor detection beyond OSNs are less explored.

Anatomically, OSNs project axons to the olfactory bulb (OB), where their axons terminate and form synapses with projection neurons and local interneurons (Buck, 1996; Lledo et al., 2005; Nagayama et al., 2014; Mori and Sakano, 2021). Each OSN expresses a single type of ORs out of a repertoire of ∼1,100 members in rodents and ∼400 in humans (Buck and Axel, 1991). OSNs expressing the same type of ORs project their axons precisely and reproducibly to a pair or few of glomeruli located on the medial and lateral sides of each OB (Ressler et al., 1994; Vassar et al., 1994; Mombaerts et al., 1996). These glomeruli are simultaneously activated by the same odorants, thus termed mirror or isofunctional glomeruli (Rubin and Katz, 1999; Lodovichi et al., 2003; Zou et al., 2009). Since its discovery decades ago, the physiological significance of this unique structural arrangement has remained unclear.

Prior to the experimental demonstration of mirror glomeruli, a neural circuit termed intrabulbar associational system (IAS) reciprocally connecting the medial and lateral sides of each OB was anatomically characterized in hamsters (Schoenfeld et al., 1985). IAS is predominantly formed by a subpopulation of tufted cells (TCs) with somata located in the superficial portion of the external plexiform layer (EPL), thus called superficial tufted cells (STCs). These STCs are topographically organized to have single apical dendrites ramifying in individual glomeruli and axons projecting to the internal plexiform layer (IPL) where they course abruptly and travel along to the opposite side of the same OB terminating in the IPL right beneath the mirror glomeruli. This STC-formed unique neural circuitry was subsequently demonstrated in rats and mice to link mirror glomeruli (Liu and Shipley, 1994; Belluscio et al., 2002; Lodovichi et al., 2003). Furthermore, the IAS refinement exhibits a high level of activity-dependent plasticity (Marks et al., 2006; Cummings and Belluscio, 2010), indicating active IAS participation in olfactory processing. Thus, elucidating the functional mechanisms underlying the IAS operation will potentially shed light on the biological significance of mirror glomeruli organization in the olfactory system.

To explore the principles governing the functional operation of IAS, it is important to understand the synaptic interactions between the IAS-forming STCs and the circuit-related neurons. Since the IAS-forming STCs exclusively express the neuropeptide cholecystokinin (CCK; Liu and Shipley, 1994), this enables us to utilize it as a molecular marker to selectively label and manipulate activities of these cells and the IAS circuit with optogenetic and chemogenetic approaches and analyze the functional readout of other local neurons and animals' behavioral outcome. For example, recent studies with these approaches have revealed that IAS-forming STCs actively recruit local GABAergic interneurons (Sun et al., 2020) and participate in odor detection (Chen et al., 2024). However, the cellular and circuit mechanisms underlying the roles of these CCKergic tufted cells in odor detection remain elusive. Given the ramification in individual glomeruli by apical dendrites of both CCKergic STCs and mitral cells (MCs), we hypothesize that CCKergic STCs interact with MCs and modulate their output. To test this, we designed and completed a series of experiments with a clear demonstration of CCKergic TC's role in intermediating feedforward monosynaptic excitation to MCs on both sides of each OB to regulate OB output to downstream centers, thus enabling the system to detect weak signals by increasing odor detection sensitivity at the cellular and circuit levels in the OB.

## Materials and Methods

### Animals

Wild-type (C57BL/6J) and transgenic, homozygous CCK-Cre mice were obtained from The Jackson Laboratory. Homozygous CCK-Cre mice (CCK-Cre+/+) were maintained by breeding male and female CCK-Cre+/+ mice. Heterozygous CCK-Cre (CCK-Cre+/−) mice were generated by breeding male homozygous CCK-Cre mice and female wild-type C57BL/6J mice. Animals were maintained with a standard 12 h light/dark cycle with water and food *ad libitum*. All experimental procedures were performed in accordance with approval from the Institutional Animal Care and Use Committee of Howard University and the University of Georgia.

### Surgical procedures and viral injection

Stereotaxic viral injections were performed as previously described (Sun et al., 2020). Briefly, animals were deeply anesthetized by an initial intraperitoneal (i.p.) dose of 100 mg/kg ketamine and 10 mg/kg xylazine mixture before the skull was exposed and a craniotomy (1 mm) was drilled on the midline between two bulbs or 1.2–1.3 mm lateral from the midline at 3.95 mm from Bregma. For all experiments, injections were localized to the superficial EPL of the OB. The coordinates for injection in the medial OB were 0.3 mm from the midline and 1.5 mm depth from the OB dorsal surface. For the lateral OB injection, the coordinates were 1.2–1.3 mm from the midline at a depth of 1.2–1.3 mm from the OB dorsal surface. For tufted cell labeling or electrophysiological experiments, mice (4–6 weeks of age) were injected in the superficial EPL on the medial side of the OB with one of the following: adeno-associated virus (AAV) serotype 5 carrying fusion genes for the fluorescent protein tdTomato, channelrhodopsin 2 and enhanced yellow fluorescent protein (AAV5-ChR2-EYFP), or halorhodopsin (eNpHR 3.0) and EYFP (AAV-HR-EYFP). For behavioral experiments, we utilized the designer receptors exclusively activated by designer drugs (DREADs). Mice (4–5 weeks) were injected with AAV5 carrying fusion gene encoding the inhibitory DREADD hM4D(Gi) and the fluorescent protein mCherry into both medial and lateral sides of the OB (AAV-hM4D_i_-mCherry). AAV injections were conducted by a nanoliter injector (Nanoject III, Drummond Scientific) at a rate of 10 nl/s. All animals recovered for 4–6 weeks to ensure viral expression prior to the onset of experiments. All viruses were purchased from Addgene.

### Slice preparation

For electrophysiological experiments, OB slices were obtained from 8–10-week-old male and female mice. Animals were anesthetized with isoflurane prior to decapitation, and OBs were rapidly dissected and mounted onto a VT1200S Vibratome disc (Leica) using superglue. Horizontal or coronal OB slices (350 µM) were cut in oxygenated (95% O_2_–5% CO_2_) ice-cold sucrose–ACSF containing the following (in mM): 210 sucrose, 2.0 KCl, 1.25 NAH_2_PO_4_, 26 NaHCO_3_, 10 glucose, 8 MgSO_4_, and 0.5 CaCl_2_. Slices were immediately transferred to and incubated in continuously oxygenated ACSF which were heated to 30°C and had the following composition (in mM): 124 NaCl, 2.5 KCl, 1.25 NaH_2_PO_4_, 6.0 MgSO_4_, 2.0 CaCl_2_, 26 NaHCO_3_, and 10 glucose. Following incubation, slices were transferred to and maintained for at least 30 min at room temperature (RT) in normal ACSF containing (in mM) 124 NaCl, 2.5 KCl, 1.25 NaH_2_PO_4_, 2.0 MgSO_4_, 2.0 CaCl_2_, 26 NaHCO_3_, and 10 glucose, before being used for recordings. In the recording chamber, slices were perfused at 3 ml/min with continuously oxygenated ACSF warmed to 30°C.

### Electrophysiology

Whole-cell patch-clamp recordings were made from OB neurons visualized via an Axio Examiner.A1 (Zeiss) upright epifluorescence microscope equipped with near-infrared differential interference contrast optics. Visualization of neurons was obtained by loading patch pipettes with 5 µM Alexa Fluor 594 (AF 594). Mitral cells (MCs) were identified by their somatic localization to the mitral cell layer and the presence of their apical and lateral dendrites projecting into the EPL (Macrides and Schneider, 1982; Burton and Urban, 2014; Liu et al., 2016). STCs were identified by their somatic localization within the superficial EPL and the presence of one or more lateral dendrites (Liu and Shipley, 1994; Sun et al., 2020). For post hoc reconstruction of the recorded neurons, 0.2% neurobiotin or biocytin was included in the patch pipette solution.

Current and voltage signals were recorded with a MultiClamp 700B amplifier (Molecular Devices) and low-pass filtered at 4 kHz and sampled at 10 kHz with a Digidata 1550B 16-bit analog-to-digital converter using Clampex 11.1 software (Molecular Devices). Patch recording electrodes (4–10 MΩ) were pulled from thin-wall glass capillary tubes with filament (Sutter Instrument). For current-clamp whole-cell recording, patch pipettes contained the following (in mM): 122 K-gluconate, 4 EGTA, 0.5 CaCl_2_, 5 MgCl_2_, 10 HEPES, 3 Na_2_-ATP, 0.3 Na_3_-GTP, and 10 tris-phosphocreatine (285–295 mOsm, pH 7.27 with KOH). In some cases, a depolarizing current was injected to maintain a recorded MC at a membrane potential near the action potential threshold. To record spontaneous and evoked excitatory postsynaptic currents (EPSCs), patch pipettes were filled with a recording solution containing the following (in mM): 133 CsCH_3_O_3_S, 3 EGTA, 0.4 CaCl_2_, 5 QX-314, 4 MgCl_2_, 10 HEPES, 3 Na_2_-ATP, and 0.3 Na_3_-GTP (285–295 mOsm, pH 7.27 with CsOH). All cells were held at −50 mV to permit the study of both NMDAR- and AMPAR-mediated synaptic events.

### Electrical and optical stimulation

Electrical stimulation of the olfactory nerve (ON) was delivered by a bipolar stimulation electrode, which was made from theta borosilicate tubes and filled with ACSF. Isolated and constant current pulses (0.1 ms) were triggered by a Master-9 pulse stimulator with an ISO-Flex stimulus isolator (AMPI). Optical stimulation was produced by a Polygon 400E illuminator (Mightex), which enables localized stimulation to distinct regions of the OB. Both the size of the optic window and the intensity of the optic stimulation were coordinated via PolyScan software (Mightex). The size of the optic window varied depending on experiments, ranging from 30 µM diameter for internal plexiform stimulation (IPL) stimulation to 90 µM diameter for glomerular stimulation. Validation of lack of ChR2 expression on MCs was performed by setting a circular optic window over the cell body alone (20–30 µM in diameter), and cells exhibiting responses following optic stimulation (1 ms) were interpreted as internal tufted cells and thus were discarded from the final results. For optical stimulation of STC apical dendrites, a circular optic window (diameter of 90 µM) was placed at glomeruli localized containing the apical dendrites of a recorded MC. Finally, for IPL stimulation, a circular optic window was placed directly under the recorded MC (diameter, 30 µM) and spanning from the superficial IPL to the deep IPL. The onset and duration of optical stimulation were measured by the same MultiClamp 700B amplifier and monitored throughout the duration of the experiment.

### Drug application

All drugs, unless noted otherwise, were bath applied during experiments. d-2-Amino-5-phosphonopentanoic acid sodium salt (APV, 50 µM) and 6,7-dinitroquinoxaline-2,3-dione disodium salt (DNQX, 20 µM) were purchased from Tocris Cookson. Clozapine-*N*-oxide (CNO) dihydrochloride was purchased from Hello Bio and dissolved in deionized (DI) water as a stock solution before being diluted to final concentration with ACSF or sterile saline for bath application (10 µM) in electrophysiological experiments or intraperitoneal injections (3 mg/kg body weight) for in vivo behavioral studies, respectively. Given its elimination half-life of ∼7.5 h (Guitton et al., 1998; Allen et al., 2019; Jendryka et al., 2019), animals received a dose of CNO ip injection every 8 h in the two-bottle discrimination test as described below. In other behavioral tests, a single CNO injection was delivered 30 min before the test started (Jendryka et al., 2019). Neurobiotin was purchased from Vector Laboratories. CY3-conjugated to streptavidin was purchased from the Jackson ImmunoResearch Laboratories. Alexa Fluor 594 hydrazide was purchased from Thermo Fisher Scientific. All other drugs were purchased from Sigma-Aldrich. All drugs were dissolved in DI water as stock solutions and diluted in ACSF to their final concentrations.

### Behavioral studies

#### Buried food test

This test is to assess the animal's detection of food odors (Yang and Crawley, 2009; Machado et al., 2018). In 2 consecutive days prior to the test, animals were individually housed with a piece of cookie (∼1 g) placed in their cages for them to consume. Then mice were food-deprived for 24 h before the test, during which a mouse was placed in a clean test cage containing 1 inch of clean bedding material to acclimate for 10 min. After the mouse was removed from the test cage, a piece of cookie was buried at a random location under the bottom of the bedding material. The test started by reintroducing the mouse to the test cage and recording the time taken for the animal to retrieve the cookie. For those failing to identify the buried cookie in 10 min, 600 s was assigned as their latency score. All animals foraged for food by actively searching and digging in the test cage, suggesting that fasting sufficiently motivates animals to perform the test. As the control experiment testing animal’s visual ability to identify the food source, the latency for each animal to identify the visible cookie placed on the top of bedding material in the same test cage was also recorded at the end of each BFT experiment.

#### Two-bottle discrimination test

As previously described (Wysocki et al., 1977; Pourtier and Sicard, 1990; Griff and Reed, 1995), this assay measures the animal’s ability to detect the presence of a monomolecular odorant at progressively decreased concentrations in the drinking solution and thus is considered as an odor detection threshold test. Briefly, mice were singly housed with no water supply but restricted access to a bottle of saccharin–phthalic acid solution (SPS, 2.1 × 10^−2^ M sodium saccharin and 10^−3^ M phthalic acid) for 1 h twice a day for 2 consecutive days to ensure that they would start drinking when the solution bottle was available. On the third day, animals were given a bottle containing SPS with 10^−3^ M isovaleric acid (iVA), which has a robustly distinctive odor, for 10 min. Immediately after exposure to and drinking iVA-containing SPS, each mouse received a single dose (15 µl/g body weight) of intraperitoneal (i.p.) injection of 0.6 M LiCl to induce an aversive state and was returned to a clean cage. Two hours later, each mouse was provided two bottles, one with SPS and the other with the same solution containing 10^−3^ M iVA. For every 24 h, the fluid consumption amount from each bottle by individual animal was determined by weighing bottles followed by iVA concentration reduction by 10-fold/24 h from 10^−3^ M to 10^−7^ M as the experiment continued for 5 consecutive days. The positions of the odorized versus nonodorized bottles in each cage were reversed every 12 h to minimize confound effects due to animal’s location preference or memory. An index of preference to the odorized solution for each iVA concentration was calculated as the amount of the iVA-odorized solution consumed divided by the total amount of liquid consumed for each mouse over the corresponding 24 h test period.

#### Open field test

This behavioral paradigm is based on the idea that mice naturally prefer to explore near a protective wall rather than an open area to be exposed to potential dangers and thus is utilized to assess animal's locomotion activities and anxious behavior (Seibenhener and Wooten, 2015). A square transparent test box (60 cm × 60 cm × 25 cm) is made out of plexiglass with a digital center area (30 cm × 30 cm) drawn in the video tracking program ANY-maze. Each animal was introduced to the box center followed by a video recording for 10 min. Overall activity in the test box, the amount of time, and the distance traveled in the center area of the test box were measured and analyzed.

### Immunohistochemistry

To examine tdTomato, ChR2-YFP, hM_4_D(Gi)-mCherry, or HR-YFP expression in the OB at 3–4 weeks after AAV microinjection, mice were anesthetized and transcardially perfused with 4% paraformaldehyde (PFA) before the OB tissue was prepared for immunohistochemistry as described previously (Liu et al., 2013; Liu and Liu, 2018). Whole OBs were dissected out and kept in 4% PFA at 4°C overnight. PFA was replaced in <24 h after perfusion with phosphate-buffered saline (PBS). For additional preservation of OBs for >7 d, PBS was replaced with a long-term protectant solution containing X. Coronal OB sections (50 µm thickness) were cut using a Compresstome VF-310-0Z (Precisionary Instruments) and kept in PBS at room temperature (RT). OB sections were then transferred to six-well culture dishes and rinsed with PBS three times (5 min/each) after gentle shaking. Slices were then incubated in a 0.1 M PB solution containing 4′,6-diamidino-2-phenylindole (DAPI; 5 μg/ml) at room temperature in the dark for 10 min with gentle shaking. After washing with PBS (5 min each) three times, sections were wet-mounted and coverslipped with fluorescence mounting media. Coverslip-mounted slides were stored at 4°C in the dark until imaging with confocal microscopy.

Horizontal or coronal OB slices (350 µm) containing neurobiotin-filled neurons in electrophysiological experiments were kept at 4% PFA at 4°C overnight. Slices were washed three times (5 min/each) with PBS and incubated for 1 h at RT with gentle shaking in a blocking solution made by PBS containing 1% (w/v) bovine serum albumin and 0.5% (v/v) Triton X-100. After being transferred to and incubated in the same blocking solution containing streptavidin–CY3 (1 μg/ml) covered with aluminum foil to prevent light exposure at RT on a shaker for 7 h, slices were washed three times in PBS (5 min/each) to rinse streptavidin–CY3. DAPI staining followed by section mounting was performed as described above.

Fluorescent images of fixed slices were captured using a Nikon Ti-E PFS (Nikon) inverted spinning disk confocal microscope equipped with 20× and 40× 1.4 NA Plan Apo Lambda objectives or a Zeiss LSM 900 confocal microscope with AI sample finder equipped with 2.5×/0.085 and 40×/1.4 objectives. The Nikon system is outfitted with a Yokogawa CSU-X1 (Yokogawa Electric) spinning disk unit, a self-contained four-line laser module (excitation at 405, 488, 561, and 640 nm), and an Andor iXon 897 EMCCD camera. The Zeiss system has four diode lasers (405, 488, 561, and 640 nm) for fluorescence excitation and four fluorescence filters for imaging (blue for DAPI, green for GFP or YFP, red or far red for mCherry, tdTomato or CY5). Fluorescence images of OB sections (50 µm) were collected and exported in TIFF file format.

### Statistical analysis

All numerical data are presented as the mean ± SEM. Both action potential and EPSC events were detected in Clampfit 11.1. The action potential detection threshold was set to −30 mV to exclude EPSP detection. Minimal membrane potential was measured as described previously (Liu and Shipley, 2008). EPSC detection threshold was set just below baseline to permit evaluation of area under the curve and amplitude of EPSCs. Group data were plotted with Origin Pro 2020 (OriginLab). The ON-evoked burst duration in the current clamp was measured as the time difference between the peak time of the first action potential and the last one in each response. Statistical significance for electrophysiological experiments (*p* < 0.05) was assessed using Student's *t* test or one-way repeated measure ANOVA with Bonferroni’s post hoc comparisons. Paired Student's tests were used for comparing data in the presence and absence of distinct drugs (DNQX, APV, and CBX) in the same populations of cells. Behavioral tests were assessed with a one-way ANOVA with Bonferroni’s post hoc comparisons.

## Results

### Predominant targeting the CCKergic superficial tufted cells

**Figure 1.**
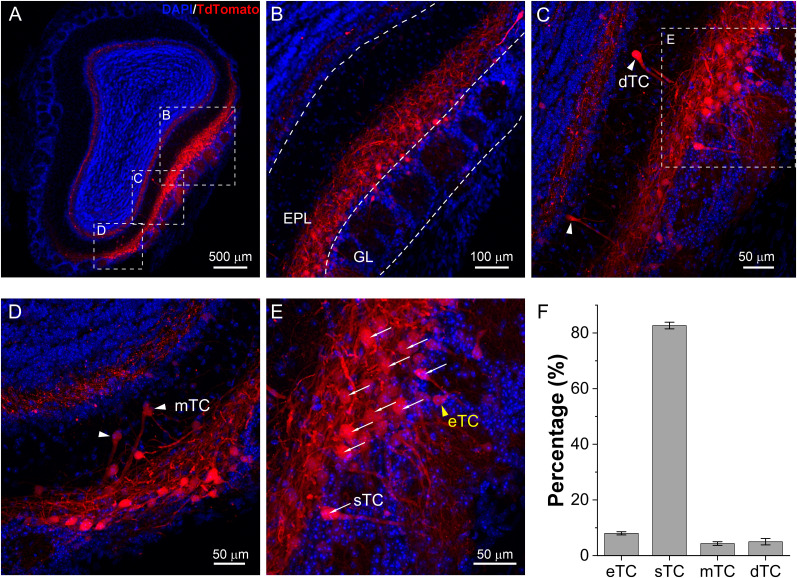
Preferential labeling of OB superficial tufted cells. ***A***, Confocal imaging of a coronal OB section from a CCK-Cre mouse 4 weeks after AAV5-FLEX-tdTomato injection into the medial side of the bulb. Predominant tdTomato fluorescence is notable in the superficial EPL on the medial side and IPL of the whole bulb. ***B***, A zoom-in photo from ***A*** showing only a few cells are labeled in the glomerular layer. ***C***, ***D***, Zoom-in photos from ***A*** highlighting examples of deep (dTC, ***C***) and middle tufted cells (mTC, ***D***) labeled. ***E***, Zoom-in photo from C showing representative labeling of superficial (sTCs) and external tufted cells (eTCs) in the EPL or the glomerular layer. ***F***, Quantified data showing the percentage of each subpopulation of tufted cells are labeled with our approach.

The neuropeptide CCK has been detected in cells of different OB layers (Seroogy et al., 1985) but with a predominant distribution in the superficial EPL in rats (Liu and Shipley, 1994). A similar distribution pattern of CCKergic neurons has been reported in the OB of transgenic CCK-Cre mice, which express the Cre recombinase under the CCK promoter, thus enabling selective labeling and optogenetic or chemogenetic manipulation of these CCK-containing neurons (Sun et al., 2020; Chen et al., 2024). Although a general consensus is that CCK is only expressed by TCs in the OB, previous studies with the same transgenic CCK-Cre mice reported a varied percentage of labeling each out of four subpopulations of TCs (Marks et al., 2006; Cheetham et al., 2015; Economo et al., 2016; Short and Wachowiak, 2019; Sun et al., 2020; Zak and Schoppa, 2021; Chen et al., 2024), which are termed as external (eTCs), superficial (sTCs), middle (mTCs), and deep tufted cells (dTCs) based on their cell body location in the OB. This variation may reflect the differences in multiple experimental variables including substrains of animals used (homozygotes vs hemizygotes), labeling approaches (animal crossing and viral transfection), animal age for virus injection, and viral serotypes. Our previous work showed a predominant labeling of superficial TCs in the EPL with a viral transfection approach (Sun et al., 2020). However, due to our lack of quantifying eTCs, a subpopulation of TCs providing direct feedforward excitation to MCs (De Saint Jan et al., 2009; Najac et al., 2011; Gire et al., 2012) and exhibiting a remarkable labeling in a recent study with the same animal model (Zak and Schoppa, 2021), we performed this experiment to reassess the relative labeling of four different subpopulations of TCs including eTCs. Since TCs are mainly classified by their soma location, we chose Cre-dependent AAV5-FLEX-tdTomato to target CCKergic cells due to its preferential labeling of neuronal somata via cytosolic Cre. As shown in Figure 1, *A* and *B*, the OB superficial EPL and IPL were intensely labeled by tdTomato 3 weeks after virus injection. At higher magnification, dTCs (Fig. 1*C*), mTCs (Fig. 1*D*), and sTCs in the EPL (Fig. 1*E*) were easily identifiable and quantified purely based on their soma location in the EPL. However, the distinguishment between sTCs and eTCs at the boundary between the EPL and glomerular layer (GL) was challenging due to the lack of solid criteria in the literature. To circumvent this, we established the following three morphological criteria for categorization and quantification of them. (1) Soma size: compared with sTCs (>15 µm diameter), eTCs have relatively small somas (<15 µm diameter). (2) Length of the initial unbranched apical dendrite: sTCs have relatively long apical dendrites with branches distal to the soma while eTCs have short apical dendrites with branches proximal to the soma. By measuring from the soma center to the first branch: sTC (>30 µm) and eTC (<30 µm). (3) Soma shape and presence or absence of lateral dendrites: eTCs have pear-shaped somas with a round and smooth base but no lateral dendrites while sTCs have lateral dendrites or protuberance(s) on the somatic base even when the lateral dendrites are truncated during slicing. Thus, we classified those with relatively large soma and long unbranched initial apical dendrites but with protuberances at the soma base as sTC as shown in Figure 1*E*. Based on these classification criteria, our analysis of data from OB sections collected from three mice showed that a vast majority (82.7%) of labeled cells were sTCs (248/300 cells), 8% (24/300 cells) eTCs, 4.3% (13/300 cells) mTCs, and 5% (15/300 cells) dTCs (Fig. 1*F*). This suggests that our approaches for labeling and opto- and chemogenetic manipulations preferentially target CCKergic STCs.

### Contribution of AMPA and NMDA receptor activation to the ON-evoked STC responses

**Figure 2.**
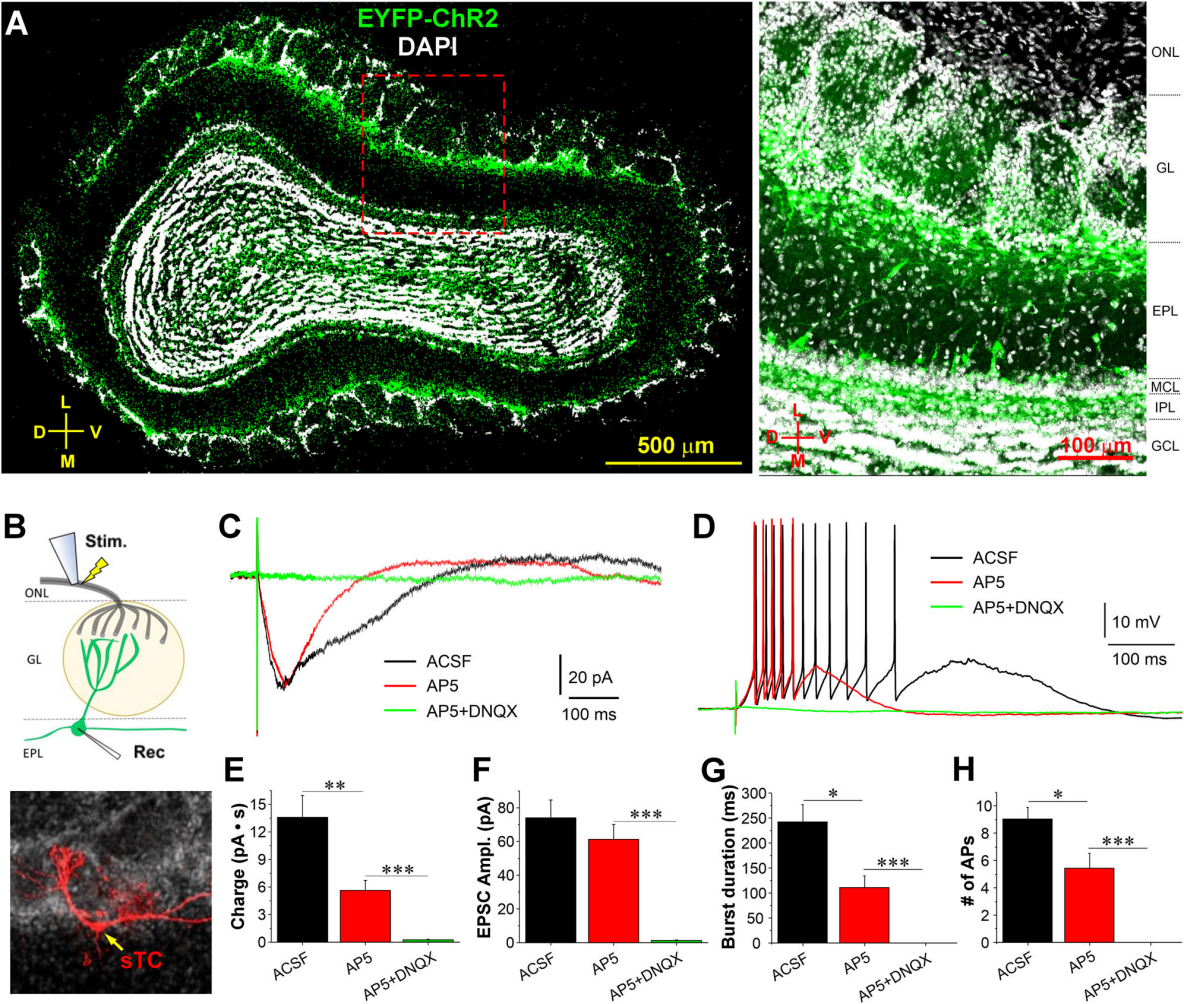
AMPA and NMDA receptors mediate the ON-evoked CCKergic STC response. ***A***, Left, Confocal image of a coronal OB section showing the expression of ChR2-EYFP (green) predominantly in the CCKergic STCs with somata located in the superficial EPL, apical dendrites ramifying in individual glomeruli, and axons traveling and confined to the IPL. The section was counterstained with DAPI (white). Right, Blown-up photo from ***A*** to highlight ChR2-EYFP expression in the different layers. D, dorsal; V, ventral; M, medial; L, lateral; EPL, external plexiform layer; GCL, granule cell layer; GL, glomerular layer; IPL, internal plexiform layer; MCL, mitral cell layer; ONL, olfactory nerve layer. ***B***, Top, Schematic of experimental design. Bottom, Confocal image of a typical sTC (red) recorded and filled with biocytin reconstructed by immunohistochemical staining. Note a single primary/apical dendrite ramifying in one glomerulus and a secondary dendrite running laterally. ***C***, ***D***, Typical voltage-clamp (***C***) or current-clamp (***D***) traces recorded from an EYFP-labeled CCKergic STC showing the ON-evoked responses in ACSF (black), in the presence of APV (red) or DNQX and APV (green). ***E***–***H***, Bar graphs representing quantified data of the ON-evoked responses in the CCKergic STCs in terms of charge or amplitude (***F***) of EPSCs, burst duration (***G***), or number of action potentials (APs, ***H***) in ACSF or in the presence of APV or APV and DNQX. *p < 0.05; **p < 0.01; ***p < 0.001.

Our previous study showed that the ON-evoked responses in CCKergic STCs were completely blocked by the mixture of selective AMPA receptor blocker NBQX and NMDA receptor blocker APV. However, the relative contribution of each receptor type is unknown. To address this question, we conducted both voltage- and current-clamp recordings of ON-evoked responses in CCKergic STCs in OB slices prepared from CCK-Cre mice (Fig. 2*A*). CCKergic STCs were identified for recording by their cell body location in the superficial EPL and expression of ChR2-EYFP (Fig. 2*B*). Consistent with our previous study, electrical ON stimulation elicited excitatory postsynaptic currents (EPSCs; Fig. 2*C*, black trace) or a burst of action potentials (APs; Fig. 2*D*, black trace) in the recorded STCs when they were voltage clamped at −60 mV or in the current clamp and perfused with normal ACSF. The duration of these responses was significantly reduced by the selective NMDA receptor blocker D-APV (50 µM; Fig. 2*C*,*D*, red traces). The response charge (time integral) of ON-evoked EPSCs was decreased by 57.6% from 13.59 ± 2.38 to 5.76 ± 1.06 pA·s (*n* = 5 cells, *t*_(8)_ = 4.33427, *p* = 0.00749, one-way RM ANOVA with Bonferroni’s comparison; Fig. 2*E*) while EPSC amplitude was not significantly changed by D-APV with 74.1 ± 10.6 pA in ACSF versus 61.3 ± 8.8 pA in D-APV (*n* = 5 cells, *t*_(8)_ = 1.55357, *p* = 0.45401, one-way RM ANOVA with Bonferroni’s comparison; Fig. 2*F*). Congruently, the ON-evoked burst duration, the peak time stamp difference between the first and last action potentials, was reduced by 54% from 241.4 ± 35.5 to 110.9 ± 22.5 ms (*n* = 5 cells, *t*_(8)_ = 4.01954, *p* = 0.01153, noe-way RM ANOVA with Bonferroni’s comparison) whereas the number of action potentials (APs)/response decreased by 39.7% from 9.04 ± 0.89 to 5.45 ± 1.07 (*n* = 5 cells, *t*_(8)_ = 3.86459, *p* = 0.01433, one-way RM ANOVA with Bonferroni’s comparison). The residual components of ON-evoked responses were completely blocked by the combination of selective AMPA and NMDA receptor blockers DNQX (20 µM) and D-APV (50 µM; Fig. 2*C*–*H*). These findings demonstrate that NMDA receptors in STCs actively participate and amplify the ON-elicited postsynaptic responses.

### STCs provide excitatory feedforward input to mitral cells via their apical dendrites

**Figure 3.**
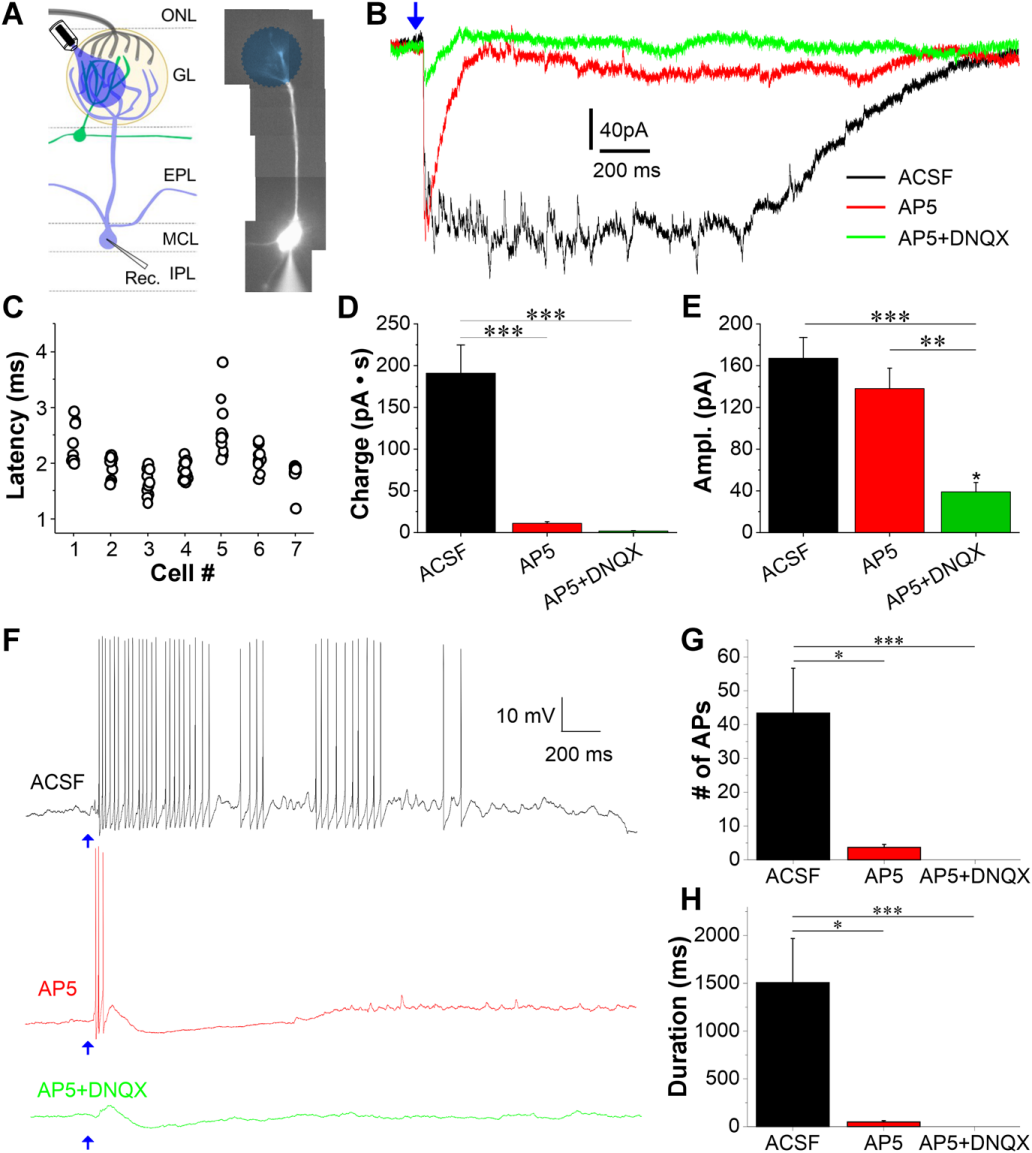
CCKergic STCs provide long-lasting excitation to MCs via dendrodendritic transmission in the glomerulus. ***A***, Left, Illustration of experimental design. Right, Photo of a recorded mitral cell filled by the fluorescent dye Alexa Fluor 594 via the patch-clamp recording pipette. EPL, external plexiform layer; GL, glomerular layer; IPL, internal plexiform layer; MCL, mitral cell layer; ONL, olfactory nerve layer. ***B***, Representative voltage-clamp recording traces showing the long-lasting MC response to optogenetic activation of CCKergic STC apical dendrites in the glomerulus in ACSF (black) or after bath application of APV (red) or APV and DNQX (green). Blue arrow denoting blue light stimulation. ***C***–***E***, Pooled data of seven cells showing the onset latencies (***C***), charge (***D***), or initial peak amplitude (***E***) of the STC-evoked MC responses under different conditions as shown in ***B***. ***F***, Representative current-clamp recording traces showing the long-lasting MC response to optogenetic activation of CCKergic STC apical dendrites in the glomerulus in ACSF (black) or after bath application of APV (red) or APV and DNQX (green). ***G***, ***H***, Pooled data of five cells showing the number of action potentials (***G***) or spike burst duration (***H***) of the STC-evoked MC responses under different conditions as shown in ***F***. *p < 0.05; **p < 0.01; ***p < 0.001.

Previous studies demonstrate that single apical dendrites of STCs ramify in individual glomeruli (Orona et al., 1984; Liu and Shipley, 1994; Sun et al., 2020) where they potentially establish synaptic connections with local interneurons and/or apical dendrites of mitral/tufted cells. Actually, STCs form dendrodendritic synapses with two populations of GABAergic interneurons short axon cells (SACs) and periglomerular cells, highlighting the role of STCs in coordinating inhibitory circuits in the glomerular layer (Sun et al., 2020). A recent study reported that optogenetic silencing of CCKergic cells can reduce MC excitatory responses evoked by OSN stimulation (Zak and Schoppa, 2021), indicating the participation of CCKergic neurons in the ON-evoked MC output. However, direct evidence supporting the CCKergic TC→MC transmission is lacking. To examine potential dendrodendritic synaptic transmission from STCs to MCs in the glomerulus, recordings were made from MCs on the medial side of OB slices prepared from CCK-Cre mice with ChR2 expression in CCKergic STCs as shown in Figure 2*A*. Alexa Fluor 594 (10 µM) was included in the internal solution to visualize MC apical dendrites, thus guiding the delivery of LED blue light (470 nm) to the targeted glomerulus for optogenetic activation of CCKergic STC apical dendrites (Fig. 3*A*). The circular area of blue light (∼50–90 µm in diameter) was configured and generated by a Polygon 400E and presented through the microscopic objective lens to activate ChR2 expressed on CCKergic STC apical dendrites. Brief (1 ms) optical stimulation reliably (20/20 traces in each cell) evoked long-lasting inward currents in the recorded MCs (Fig. 3*B*). These responses were characterized by a short onset latency (Fig. 3*C*) and predominately mediated by NMDARs as application of APV dramatically reduced the response charge by 94.2% from 190.97 ± 33.8 to 11.05 ± 1.86 pA·s (*n* = 7 cells, *t*_(12)_ = 6.58893, *p* < 0.0001, one-way RM ANOVA with Bonferroni’s comparison) but not the amplitude of the inward current, which was 167.2 ± 19.9 pA in ACSF and 138.0 ± 19.6 pA (*n* = 7 cells, *t*_(12)_ = 1.7478, *p* = 0.31804, one-way RM ANOVA with Bonferroni’s comparison; Fig. 3*B*,*D*,*E*). These long-lasting responses have consistently short onset latencies (2.0 ± 0.1 ms, *n* = 7 cells) with an average jitter at 279.3 ± 48.8 ms, indicating a dendrodendritic monosynaptic transmission from STCs to MCs. Due to the relatively long jitter value and potential confound effects of strong optogenetic stimulation, glutamate spillover may also contribute to this response as previously reported in eTCs (Gire et al., 2019). The addition of DNQX significantly decreased the response charge and amplitude of the optical stimulation-evoked brief inward current in the presence of APV (Fig. 3*B*,*D*,*E*) but with a small residual inward current with an average charge at 1.75 ± 0.49 pA·s (*n* = 7 cells, *t*_(12)_ = 0.3404, *p* = 1 compared with APV but *t*_(12) _= 6.92933, *p* < 0.0001 compared with ACSF, one-way RM ANOVA with Bonferroni’s comparison) and amplitude at 39.0 ± 9.0 pA (*n* = 7 cells, *t*_(12)_ = 5.94059, *p* = 0.000204353 compared with APV and *t*_(12)_ = 7.68839, *p* < 0.0001 compared with ACSF, one-way RM ANOVA with Bonferroni’s comparison; Fig. 3*B*, green trace; Fig. 3*D*,*E*), indicating that AMPA receptors mediate vast majority of these brief responses. Likewise, the same optical stimulation in the current clamp reliably evoked long-lasting depolarization (LLD) superimposed by bursts of APs (Fig. 3*F*), which have an averaged spikes/response in five cells at 43.4 ± 13.2 and burst duration at 1,507.1 ± 461.7 ms in ACSF. These values were respectively reduced by bath-applied APV by 91.5% to 3.7 ± 0.9 (Fig. 3*G*, *n* = 5, *t*_(8)_ = 3.6707, *p* = 0.01891 compared with ACSF, one-way RM ANOVA with Bonferroni’s comparison) and by 96.8% to 48.8 ± 13.7 ms (Fig. 3*H*, *n* = 5, *t*_(8)_ = 3.87493, *p* = 0.01413 compared with ACSF, one-way RM ANOVA with Bonferroni’s comparison) whereas addition of DNQX abolished all the remaining evoked spikes (Fig. 3*F*–*H*). Collectively, these findings establish that apical dendrites of STCs and MCs form glutamatergic connections in the glomerulus where STCs provide robust feedforward excitation to reliably evoke LLDs in MCs. These STC-evoked responses are predominantly mediated by the activation of postsynaptic NMDARs and AMPARs in MCs.

### STCs amplify the ON input-evoked mitral cell responses

**Figure 4.**
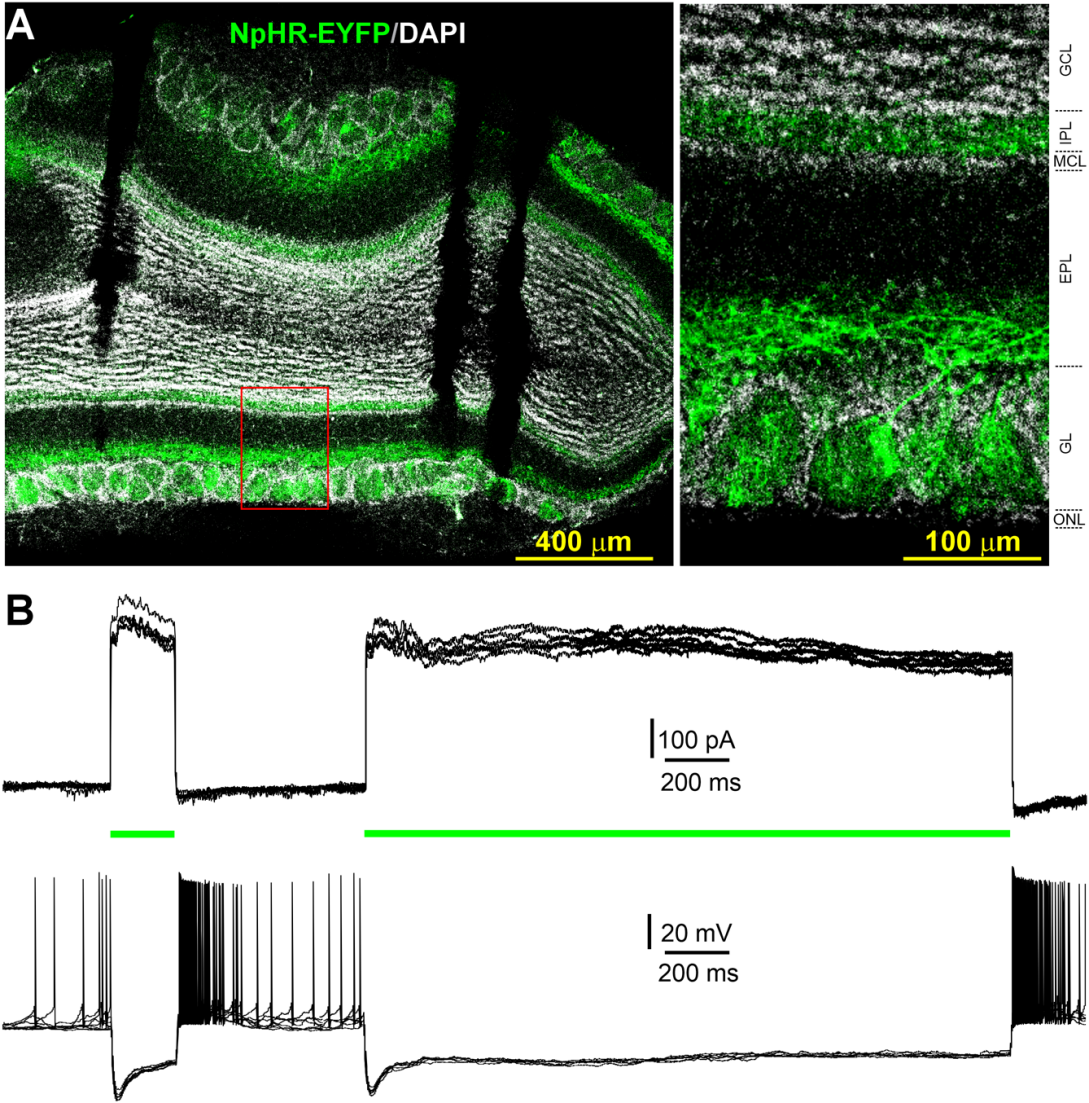
Expression and function of halorhodopsin (NpHR) in CCKergic STCs. ***A***, Left, Confocal image of a horizontal OB section showing NpHR-EYFP expression predominantly in STCs. Right, Blow-up of the area within the red square from the left. EPL, external plexiform layer; GCL, granule cell layer; GL, glomerular layer; IPL, internal plexiform layer; MCL, mitral cell layer; ONL, olfactory nerve layer. ***B***, representative recording traces from an EYFP-expressing STC in voltage (top) or current (bottom) clamp showing the outward currents or hyperpolarization elicited by a 200 ms or 2 s green light (590 nm) exposure to the recorded cell body.

**Figure 5.**
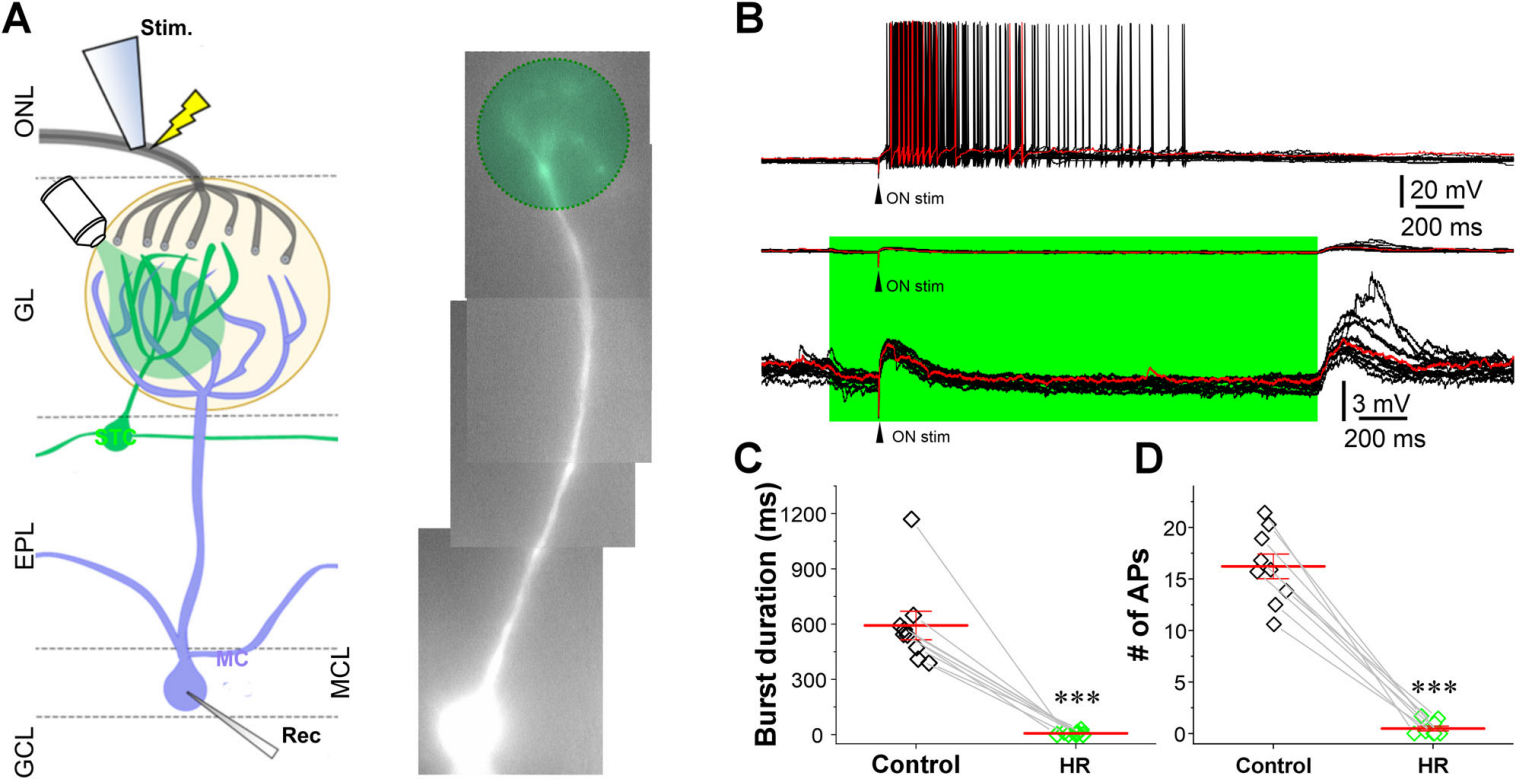
Optic inhibition of the CCKergic STC apical dendrites significantly attenuates the ON-evoked MC response. ***A***, Left, Schematic of experimental design. Right, Photo of a recorded mitral cell filled by the fluorescent dye Alexa Fluor 594 via the patch-clamp recording pipette. EPL, external plexiform layer; GCL, granule cell layer; GL, glomerular layer; IPL, internal plexiform layer; MCL, mitral cell layer; ONL, olfactory nerve layer. ***B***, Representative current-clamp traces showing MC responses to ON stimulation (black arrowhead) before (top trace) or after (middle trace) green light stimulation was turned on to activate the NpHR in STCs. Bottom trace is a blow-up of the middle trace to highlight the hyperpolarization during green light exposure. Arrowhead: olfactory nerve stimulation (ON stim). ***C***, ***D***, Data pooled from nine MCs showing the effects of light stimulation on the burst duration and number of action potentials (APs) of MC responses to ON stimulation. ***p < 0.001.

Since CCKergic STCs respond to single ON stimulation with a burst of action potentials (Fig. 2; Jones et al., 2020; Sun et al., 2020) and intermediate long-lasting feedforward excitation to MCs (Fig. 3) forming the ON→STC→MC pathway, we predict that CCKergic STCs amplify MC output in response to ON input. To test this, we expressed halorhodopsin (eNpHR) in CCKergic STCs by injecting the Cre-dependent AAV5-Ef1a-DIO-eNpHR 3.0-EYFP (AAV-HR-YFP) into the medial side of each OB of CCK-Cre mice. Similar to ChR2-EYFP expression (Fig. 2*A*), eNpHR-EYFP was predominately expressed in the superficial EPL, GL, and IPL, corresponding to the location of CCK-TC somata, apical dendrites, and axonal projections/terminations, respectively (Fig. 4*A*). This distribution of EYFP expression was consistent with the previously reported CCK expression in the OB (Seroogy et al., 1985; Liu and Shipley, 1994; Sun et al., 2020), highlighting preferential eNpHR expression in CCK-containing STCs in the OB. To verify eNpHR functionality, EYFP-expressing neurons in OB slices were recorded in the presence of APV (50 µM), DNQX (20 µM), and the selective GABA_A_ receptor blocker gabazine (10 µM). A circular green LED light (∼20 µM in diameter) was delivered to stimulate only the cell body of the recorded YFP-expressing cells. A 200 ms or 2 s green light (590 nm) stimulation reliably evoked outward currents or complete termination of spontaneous firing activities and membrane hyperpolarization, which lasted for the whole duration of light exposure, in all recorded cells (*n* = 5) that were voltage clamped at −60 mV or in the current clamp, respectively (Fig. 4*B*). No responses were observed from EYFP-negative cells on the same side or on the opposite side of the bulb (data not shown). These findings validated the effectiveness of our optogenetic approach for selective labeling and inhibiting STCs. To ensure consistency and comparability, we used the same level of light stimulation power for all remaining optogenetic experiments. To test whether CCKergic STCs play a role in amplifying the ON-evoked responses in MCs, we recorded MCs in the current clamp while ON electrical stimulation and green light optical stimulation were delivered to ON layer and the glomeruli receiving apical dendrites of the recorded MCs, respectively (Fig. 5*A*). As shown in Figure 5*B*, ON stimulation elicited a burst of action potentials in a typically recorded MC (top trace). When the green light was on, the membrane potential was hyperpolarized in the recorded MCs during the whole period of light exposure (Fig. 5*B*, middle and bottom traces). However, the same ON stimulation applied in the presence of green light exposure evoked no action potentials in the recorded MC (Fig. 5*B*, middle and bottom traces). These findings were replicated in all recorded MCs (*n* = 7; Fig. 5*C*,*D*). Specifically, ON-evoked burst duration and number of action potentials were reduced from 616.6 ± 98.8 ms and 16.9 ± 1.3 in control to 5.9 ± 3.9 ms (*n* = 7, *p* < 0.0001, paired *t* test) and 0.5 ± 0.3 (*n* = 7, *p* < 0.00001, paired *t* test) during green light exposure, respectively (Fig. 5*C*,*D*). This significantly reduced MC response to ON stimulation in the presence of green light exposure to CCKergic STCs likely reflects optogenetic inhibition of the CCKergic STC-mediated feedforward excitation to MCs. Moreover, green light produced hyperpolarization of MCs could also contribute to this effect.

### Gap junction contributes to the STC→MC transmission

**Figure 6.**
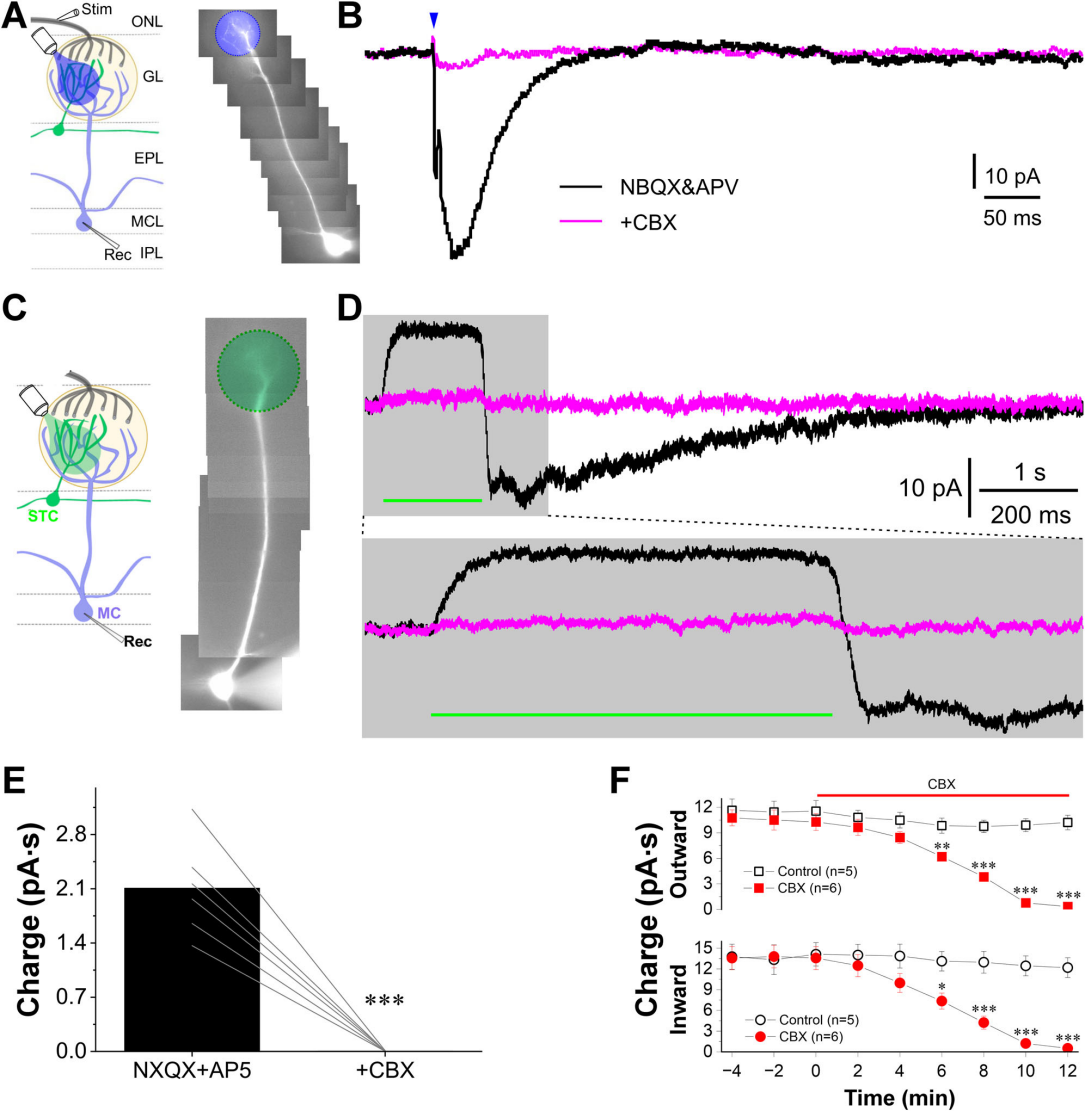
Gap junction contributes to the ON-evoked excitatory response in MCs. ***A***, ***C***, Left, Schematic illustration of the design of experiments shown in ***B*** and ***D***, respectively. Right, Photos of recorded mitral cells filled by the fluorescent dye Alexa Fluor 594 via the patch-clamp recording pipette. EPL, external plexiform layer; GCL, granule cell layer; GL, glomerular layer; IPL, internal plexiform layer; MCL, mitral cell layer; ONL, olfactory nerve layer. ***B***, Typical voltage-clamp traces showing responses of an MC in the presence of 10 µM NBQX and 50 µM APV (black) or after (purple) addition of the gap junction blocker carbenoxolone (CBX, 300 µM). ***D***, Top, Typical voltage clamp traces showing MC responses to the green light stimulation presented to activate NpHR and inhibit STC apical dendrites in the glomerulus receiving the apical dendrite of the recorded MC as shown in C in the presence of NBQX and APV (black) or with the addition of CBX (purple). Bottom, A blow-up of the response portion highlighted with gray color shown in the top panel. ***E***, Bar graphs representing quantified data comparing the ON stimulation-evoked MC responses charge in the presence of NBQX and APV (black) or with the addition of CBX (purple), respectively. ***F***, Time course effects of CBX (*n* = 6 cells) on the integrated charge of the green light-evoked both inward and outward current by comparing to control cells (*n* = 5). *p < 0.05, ***p < 0.01, ***p < 0.001.

To differentiate these possibilities, we first tested whether optogenetic inhibition of CCKergic STCs hyperpolarized MCs. Although CCKergic STCs via their apical dendrites drive MCs (Fig. 3) and GABAergic interneurons in the glomerular layer (Sun et al., 2020) which provide forward inhibition to MCs (Shao et al., 2009; Shao et al., 2012; Banerjee et al., 2015; Liu et al., 2016; Burton, 2017), selective inhibition of this subpopulation of excitatory tufted cells is not predicted to hyperpolarization of MCs unless there is a tonic excitatory driving of MCs by the CCKergic STCs or a gap junction between STCs and MCs. However, the first possibility is not supported by our finding of the synaptic blocker-resistant excitation in MCs by optogenetic activation of CCKergic STCs (Fig. 3*B*,*F*, green traces). Thus, we hypothesized the existence of gap junction-mediated electrical synapses between STCs and MCs. To test this idea, we voltage clamped MCs at −60 mV in the presence of glutamatergic synaptic blockers DNQX (10 µM) and APV (50 µM) and compared their responses to either optogenetic excitation or inhibition of CCKergic STCs before and after bath application of the gap junction blocker carbenoxolone (CBX, 300 µM). As shown in Figure 6, *A* and *B*, the MC responded with an inward current to blue light stimulation of the glomerulus receiving apical dendrite of the recorded MCs in OB slices prepared from CCK-Cre mouse with ChR2-EYFP expression in the CCKergic STCs. This response was completely eliminated by CBX treatment for 10 min. Specifically, the charge of this current in six MCs was reduced from 2.11 ± 0.25 to 0.006 ± 0.001 pA·s (*n* = 6 cells, *p* < 0.0001 paired *t* test) by CBX (Fig. 6*B*,*E*), indicating a gap junction-mediated transmission from STCs to MCs in the glomerulus.

In a separate set of experiments, MCs were voltage clamped at −60 mV in OB slices prepared from CCK-Cre mice with eNpHR expression in CCKergic STCs in the presence of DNQX and APV to block excitatory synaptic activities. When green light was presented to the glomeruli targeted by the apical dendrites of the recorded MCs to inhibit CCKergic STCs (Fig. 6*C*), robust outward currents were recorded in six out of nine MCs (Fig. 6*D*). These outward currents lasted for the whole period of green light exposure but were followed by long-lasting inward currents once the light stimulation was turned off. These green light-evoked inward and outward currents were abolished by CBX (Fig. 6*D*,*F*). The average charge of these outward and inward currents was respectively reduced from 10.5 ± 1.2 and 13.8 ± 1.6 pA·s to 0.3 ± 0.2 pA·s (*n* = 6 cell, *p* < 0.0001, paired *t* test) and 0.5 ± 0.1 pA·s (*n* = 6 cell, *p* < 0.0001, paired *t* test) after bath application of CBX for 10 min (Fig. 6*F*). To test whether the intracellular dialysis-caused rundown via the patch pipette solution contributes to the observed CBX effects, we performed another set of recordings without CBX treatment as control. The comparison of these inward and outward currents without and without CBX treatment ruled out this possibility (Fig. 6*F*). Taken together, these results demonstrate the complete elimination of bidirectional responses in MCs in response to optogenetic excitation or inhibition of CCKergic STCs by CBX strongly support gap junction-mediated electrical synapses formed between the apical dendrites of STCs and MCs in the glomerulus.

**Figure 7.**
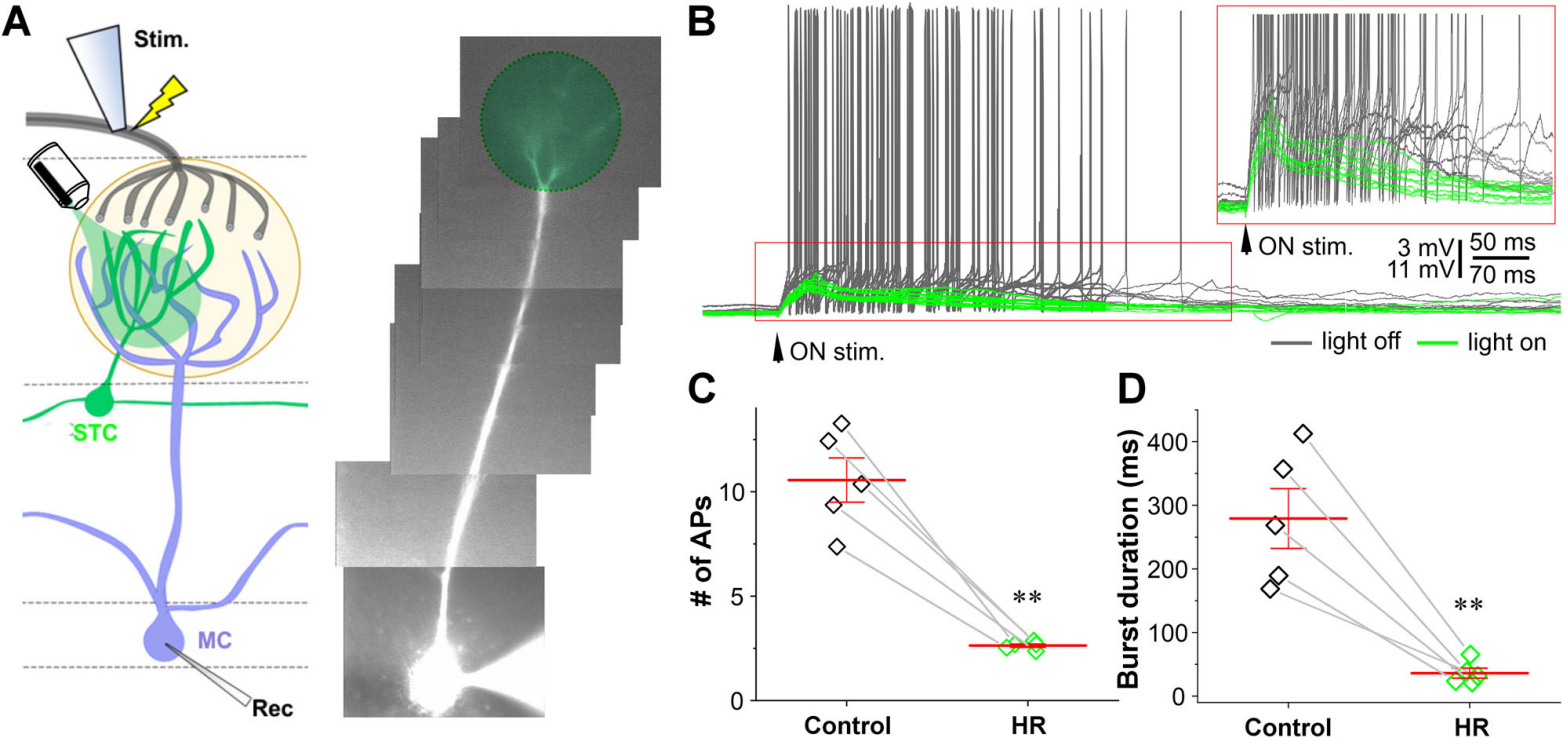
Optogenetic inhibition of CCKergic STCs drastically reduces the ON-evoked MC responses in the presence of CBX. ***A***, Left, Experimental setup. Right, Photo of a recorded mitral cell filled by the fluorescent dye Alexa Fluor 594 via the patch-clamp recording pipette. ***B***, Representative current-clamp recording traces showing MC responses to ON electrical stimulation in the absence (gray) or presence (green) of green light stimulation presented to the glomerulus affiliated by the apical dendrite of the recorded MC to activate NpHR and inhibit the CCKergic STC apical dendrites as shown in ***A***. ***C***, ***D***, Population data showing the number of action potentials (APs; ***C***) and burst duration (***D***) of the ON-evoked MC responses in the absence (control) or presence of light stimulation (HR). **p < 0.01.

To reassess STC role in intermediating ON-elicited MC responses by eliminating confound influences from gap junctions, we recorded MCs in the current clamp while ON stimulation was delivered to the olfactory nerve layer in the presence of the gap junction blocker CBX (Fig. 7*A*). Consistent with voltage-clamp results, MCs no longer responded to green light stimulation with hyperpolarization after OB slices were perfused with 300 µM CBX for 10 min (data not shown). In the presence of CBX, ON stimulation evoked a long burst of action potentials in MCs (Fig. 7*B*). However, MCs responded to the same ON stimulation with only a depolarization (EPSP) with fewer or no superimposed action potentials when the green light stimulation was turned on to selectively inhibit the CCKergic STCs affiliated with the glomeruli receiving apical dendrites of the recorded MCs (Fig. 7*B*–*D*). On the average of five cells, the ON-evoked number of action potentials (Fig. 7*C*) and burst duration (Fig. 7*D*) were reduced from 10.6 ± 1.1 and 279.2 ± 47.0 ms in the presence of CBX to 2.6 ± 0.1 (*n* = 5 cells, *p* < 0.005 paired *t* test) and 36.1 ± 7.9 ms (*n* = 5 cells, *p* < 0.005 paired *t* test) in the presence of both CBX and green light stimulation, respectively. These results support the role of the CCKergic STCs in amplifying MC output to ON input in the absence of gap junction.

In summary, these studies demonstrate that STCs provide a robust feedforward excitation to MCs via both chemical and electrical connections formed between the STC and MC apical dendrites and amplify OB output to downstream centers in response to ON stimulation.

### Stimulation of CCKergic STC axons excites MCs on the opposite side of the OB

**Figure 8.**
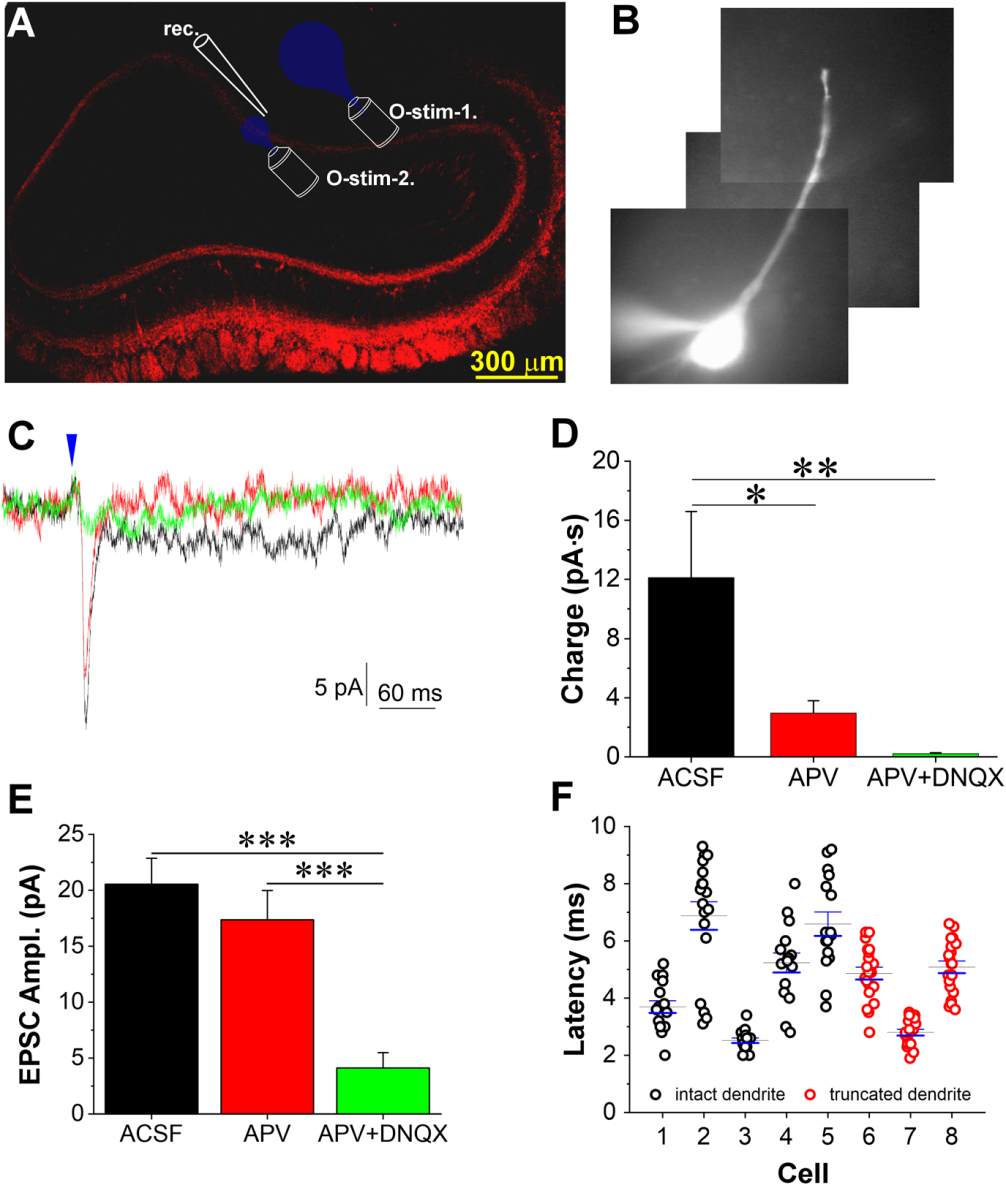
CCKergic STCs activate MCs on the opposite side of the same OB via axons in the IPL. ***A***, Illustration of experimental design on a confocal image of a coronal section of OB where ChR2-mCherry (red) was expressed in CCKergic STCs on the medial side. Note: mCherry expression only in the IPL on the lateral side, indicating axon projection from the CCKergic STCs on the medical side. ***B***, Epifluorescence microscopic photo showing a recorded MC with a truncated apical dendrite and filled by Alexa Fluor 594 via the patch pipette. ***C***, Typical voltage-clamp traces showing responses an MC on the lateral side to the optical stimulation presented to the IPL right beneath as shown in ***A*** in ACSF (black), APV (red), or APV and NBQX (green). ***D***, ***E***, Bar graphs of quantified data showing the charge (***D***) and amplitude (***E***) of EPSCs in eight MCs on the lateral side of the OB evoked by optical stimulation presented to the underneath IPL in ACSF (black), APV (red), or APV and DNQX (green). ***F***, Onset latencies of the EPSCs in eight MCs. *p < 0.05; **p < 0.01.

STC axons project to the IPL where they turn abruptly to course ventrally and dorsally to terminate in the IPL right beneath the mirror glomerulus on the opposite side of the same bulb (Liu and Shipley, 1994; Belluscio et al., 2002; Lodovichi et al., 2003; Igarashi et al., 2012). Previous studies have demonstrated that axon terminals of the CCKergic STCs provide monosynaptic inputs to GCs on the opposite side of the same OB (Liu and Shipley, 1994; Sun et al., 2020). However, whether there are functional connections formed between STC axon terminals and axon collaterals or somata of local MCs on the opposite side of the same bulb remains unknown. To answer this question, we injected 100 nl AAV into the EPL of the OB medial side of CCK-Cre mice to confine ChR2-mCherry expression only in local CCKergic STCs but not in those on the opposite side (Fig. 8*A*). In OB slices prepared from these animals, voltage-clamp recordings were made from MCs on the lateral side of the OB where ChR2-mCherry expression was detected only in the IPL but not in the superficial EPL or glomerular layer (Fig. 8*A*). To visualize the recorded cells and their apical dendrites, Alexa Fluor 594 (10 µM) was included in the recording patch pipette. No ChR2 expression in CCKergic tufted cells on the lateral side was further functionally verified by presenting the blue light to the glomeruli receiving the apical dendrites of the recorded cells (Fig. 8*A*). In these conditions, inward currents with a brief and large amplitude component followed by a slow and small amplitude component were recorded in all five MCs in response to a small sized (30 µm diameter) blue light delivered to the IPL right below the recorded cells (Fig. 8*C*) but not by a large sized (90 µm diameter) blue light presented to the glomeruli receiving their apical dendrites (Fig. 8*A*,*B*). This was an unexpected observation as no prior evidence indicates this. To further preclude potential synaptic contribution from apical dendrites in the glomeruli of the recorded cells, three MCs with apical dendrites truncated during slice preparation (Fig. 8*B*) were selected for recording but showed similar responses to optical stimulation delivered to the IPL right below somata of the recorded cells. Bath-applied APV (100 µM) completely eliminated the slow and small component of the response but only slightly reduced the amplitude of the brief component, which was significantly reduced by the addition of 20 µM DNQX. Due to no significant differences in their responses, data from the cells with intact or truncated apical dendrites were pooled together for analysis. The averaged charge of the whole response (Fig. 8*D*) and amplitude of the brief EPSC (Fig. 8*E*) in eight MCs were 12.11 ± 4.48 pA·s and 20.5 ± 2.3 pA in ACSF, 2.96 ± 0.84 pA·ms (*t*_(14)_ = 2.7193, *p* = 0.04985 compared with ACSF, RM ANOVA) and 17.4 ± 2.6 pA (*t*_(14)_ = 1.38977, *p* = 0.55892 compared with ACSF, RM ANOVA) in APV, and 0.21 ± 0.07 pA·s (*t*_(14)_ = 0.81741, *p* = 1 compared with APV; *t*_(14)_ = 3.53671, *p* = 0.00986 compared with ACSF, RM ANOVA) and 4.1 ± 1.40 pA (*t*_(14)_ = 5.79907, *p* = 0.00013812 compared with APV; *t*_(14)_ = 7.18884, *p* < 0.0001 compared with ACSF, RM ANOVA) in APV and DNQX, respectively. These results suggest a glutamatergic transmission from the CCKergic STC axons to MCs in the IPL on the opposite side of the same OB. However, the averaged onset latency of these responses in eight cells was 4.7 ± 0.6 ms with a jitter at 1,088.7 ± 208.1 µs (Fig. 8*F*). Taken together, the results of this experiment revealed a CCKergic STC-provided feedforward excitation to MCs on the opposite side of the same bulb, which is potentially polysynaptic or mediated by glutamate spillover.

### CCKergic STCs function to increase olfactory sensitivity

**Figure 9.**
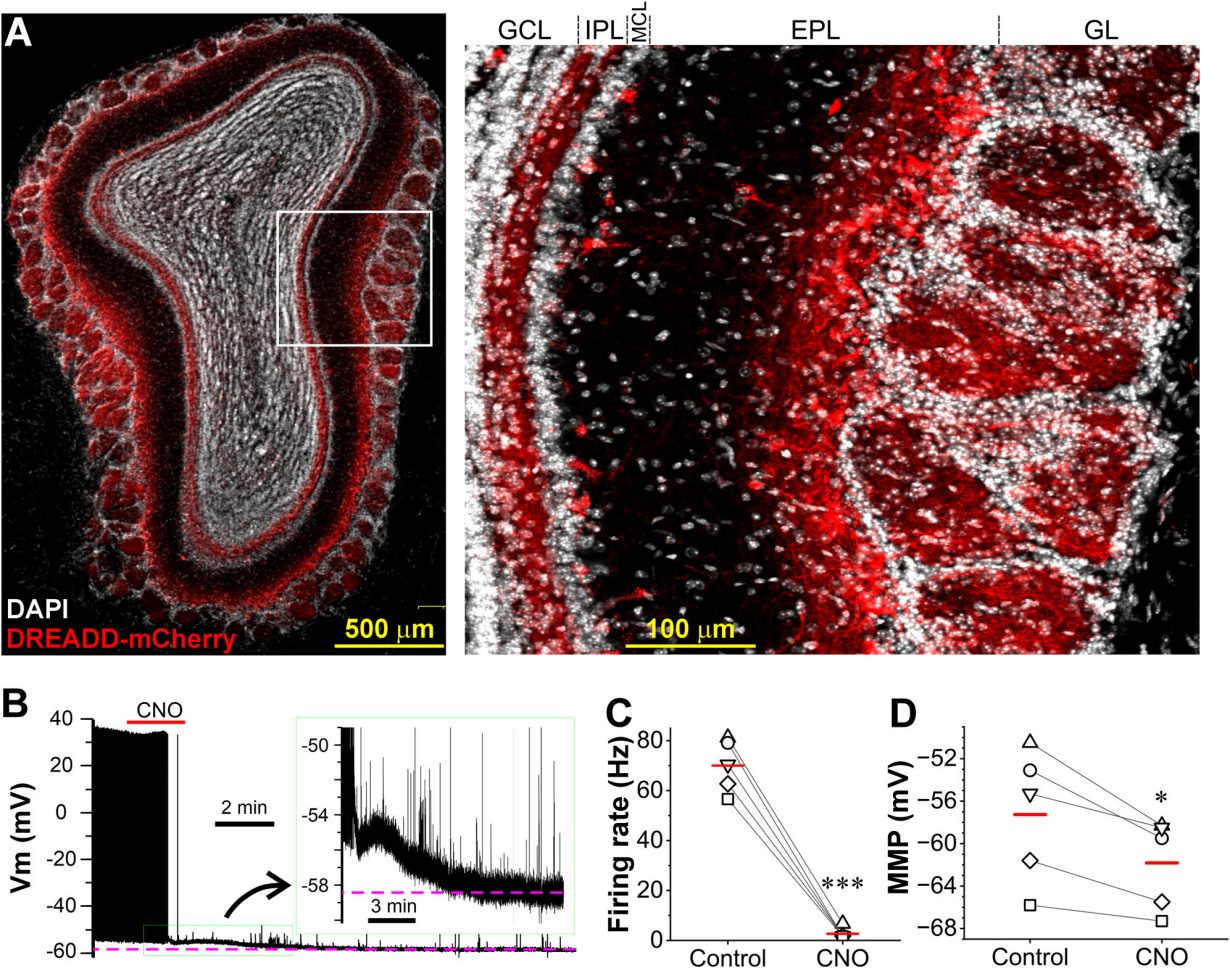
Expression and function of the inhibitory DREADD (hM4Di)-mCherry in the CCKergic STCs. ***A***, Left, Confocal image of a coronal OB section showing DREAD-mCherry (red) expression predominantly in the glomerular layer, superficial EPL, and IPL, counterstained with DAPI (white). Right, Blown-up from ***A***. EPL, external plexiform layer; GCL, granule cell layer; GL, glomerular layer; IPL, internal plexiform layer; MCL, mitral cell layer. ***B***, Typical current-clamp traces showing responses of a mCherry-expressing STC to the bath-applied DREADD actuator clozapine-*N*-oxide (CNO, 10 µM). Note: spontaneous action potentials were terminated, and the membrane was hyperpolarized by CNO as shown by the insert. ***C***, ***D***, Pooled data from five cells showing the effects of CNO on spontaneous firing rate (***C***) and minimal membrane potential (MMP, ***D***). *p < 0.05; ***p < 0.001.

The capacity for STCs to mediate MC responses to ON input via dendrodendritic transmission in the glomerulus and simultaneously excite MCs on the opposite side of the same OB in the IPL led us to hypothesize that IAS-STCs amply MC output in response to sensory input and thus function to regulate the system’s sensitivity to odorant stimuli. To test this, we compared animal’s sensitivity to odorants by two sets of behavioral tests among CCK-Cre mice that were randomly assigned to two groups with expression of either the inhibitory DREADD hMD_4_G_i_ or ChR2 in the OB CCKergic STCs. Animals in each group were further divided into two subgroups with intraperitoneal (i.p.) injection of either the hMD_4_G_i_ actuator clozapine-*N*-oxide (CNO) to activate the inhibitory DREADD in CCKergic STCs or saline 30 min before behavioral tests. Since saline does not activate hMD_4_G_i_ and neither CNO nor saline activates ChR2, CCKergic STCs were chemogenetically inhibited only in mice with hMD_4_G_i_ expression and intraperitoneal injection of CNO while mice in the other three groups including hMD_4_G_i_/saline, ChR2/CNO, and ChR2/saline, served as control animals. Thus, the comparison of behavioral performance was made among the following four groups of CCK-Cre mice to determine the functional roles of STCs: (1) AAV5-hMD_4_G_i_-mCherry injection in the OB with CNO injection (DREADD/CNO), (2) the same virus injection as in (1) but with saline injection (DREADD/saline), (3) AAV5-ChR2-mCherry injection in the OB with CNO injection (ChR2/CNO), and (4) the same virus injection but with saline injection (ChR2/saline). As shown in Figure 9*A*, the DREADD-mCherry was consistently expressed in the OB 3 weeks after virus injection with an expression pattern similar to that of ChR2 or HR. Bath-applied CNO (10 µM) hyperpolarized and terminated spontaneous action potentials in all recorded hMD_4_G_i_-mCherry-expressing neurons (in all five cells) in OB slices (Fig. 9*B*–*D*) but not in the ChR2-mCherry-expressing cells (*n* = 6 cells, data not shown). Specifically, the spontaneous firing rate was reduced from 70.0 ± 4.7 Hz in control to 2.7 ± 1.0 Hz by CNO treatment (*n* = 5 cells, *t*_(4)_ = 16.04017, *p* < 0.0001, paired *t* test) where the membrane potential was changed from −57.3 ± 2.8 to −61.8 ± 1.9 mV (*n* = 5, *t*_(4)_ = 4.03587, *p* = 0.01566, paired *t* test) by bath application of CNO. These results corroborate the effectiveness of our chemogenetic approach in inhibiting the CCKergic STCs.

**Figure 10.**
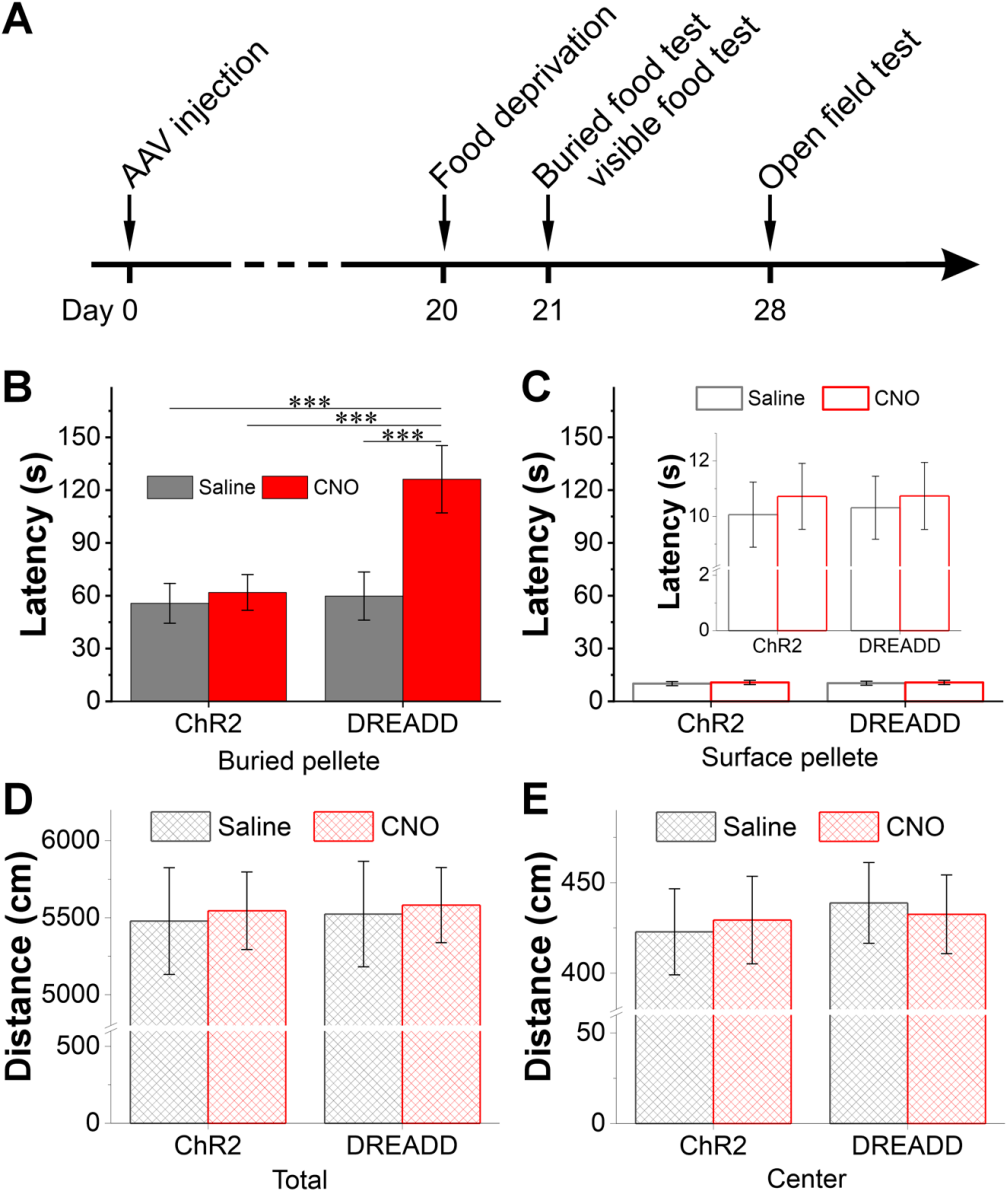
Chemogenetic inhibition of the CCKergic STCs reduces animal’s sensitivity to food odors. ***A***, Time course illustration of the experimental design. ***B***, ***C***, Bar graphs showing the latencies for identifying the buried (***B***) or visible (***C***) food in the behavioral test in four groups of mice. ***D***, ***E***, Bar graphs comparing the traveling distance in the whole testing arena (***D***) or the central zone (***E***) in the open field test behavioral test among four groups of mice. ***p < 0.001.

To investigate the functional role of OB CCKergic STCs in regulating animal’s sensitivity to odorant stimuli, we conducted the buried food test (Yang and Crawley, 2009; Machado et al., 2018), a behavioral assay for assessing olfactory detection. Three weeks after the injection of AAV5-hMD4Gi-mCherry or AAV-ChR2-mCherry (200 nl/site) into both the medial and later side of each OB, animals were subject to 23 h food deprivation before performing the behavioral test (Fig. 10*A*). As shown in Figure 10*B*, the latency (126.2 ± 61.9 s, *n* = 19) for the group of DREADD/CNO mice to identify the buried food was significantly longer than that of the other three groups, which is 59.8 ± 13.7 s (*n* = 16, *t* = 3.27579, *p* = 0.01015 compared with DREADD/CNO, two-way ANOVA) for the DREADD/saline group, 61.9 ± 10.1 s (*n* = 18, *t* = 3.27362, *p* = 0.01022 compared with DREADD/CNO, two-way ANOVA) for the ChR2/CNO group, and 55.7 ± 11.3 s (*n* = 16, *t* = 3.4793, *p* = 0.00542 compared with DREADD/CNO, two-way ANOVA) for the ChR2/saline group. In contrast, there was no difference in the latencies for animals to locate the food pellets placed on the surface of bedding material (Fig. 10*C*), indicating no contribution of visual or motor disability to the difference in buried food test results. Consistently, difference among the four groups of mice was detected in neither the total traveling distance (Fig. 10*D*) nor traveling distance in the center of the arena (Fig. 10*E*) in the open field test, suggesting that functional inhibition of the CCKergic STCs via the chemogenetic approach does not affect animal's general motor function or anxiety level. Collectively, these behavioral outcomes strongly suggest that the CCKerigic STCs function to regulate the animal's sensitivity to odor detection.

**Figure 11.**
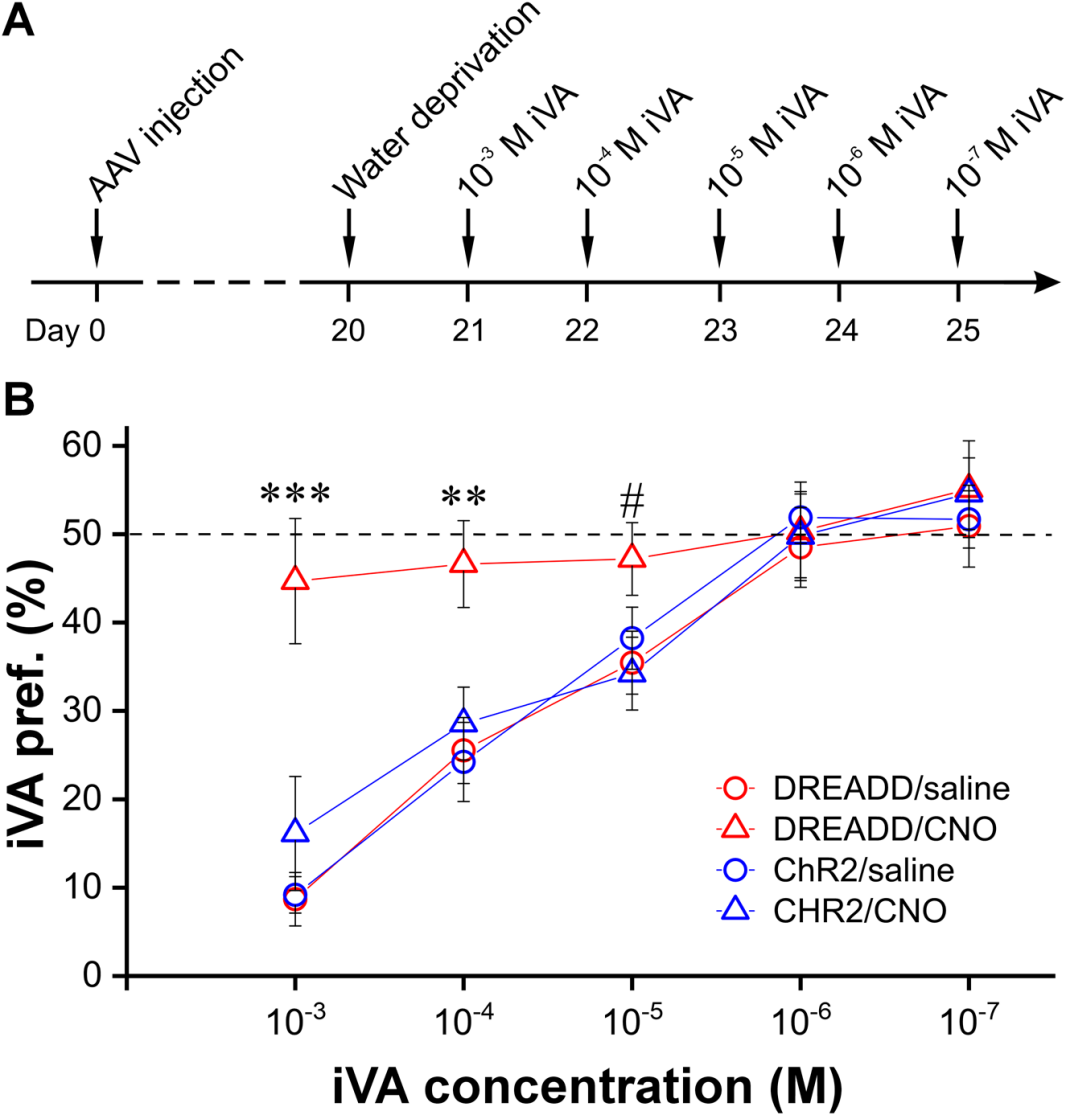
Chemogenetic inhibition of the CCKergic STCs elevates animal’s detection threshold for isovaleric acid (iVA). ***A***, Time course illustration of the experimental design. ***B***, Symbol-line graph comparing the iVA preference index among four groups of mice exposed to five decreasing concentrations (10^−3^ M to 10^−7^ M) of iVA in 5 consecutive testing days. *N* = 13 mice/group, ^#^*p* < 0.05 compared with the DREAD/saline or ChR2/CNO group, **p < 0.01 and ***p < 0.001 compared with any of the other groups.

Next, we measured the olfactory detection threshold using a two-bottle discrimination assay (Wysocki et al., 1977; Pourtier and Sicard, 1990; Griff and Reed, 1995), in which animals had a choice of drinking from two water bottles: one nonodorized and the other odorized by the monomolecular odorant isovaleric acid (iVA) at decreasing concentrations (Fig. 11*A*). Since iVA is conditioned by a single dose of intraperitoneal injection of lithium chloride (LiCl) that produces nausea-related behaviors and symptoms consistent with visceral illness in nonemetic species including mice (Ossenkopp and Eckel, 1995; Hanak et al., 2017), animals preferentially avoid drinking from the iVA-odorized water bottle until the iVA concentration decreases to or below the detection threshold. In order to motivate animals to drink from the odorized water and facilitate the LiCl-induced aversion, animals were subject to 23 h water deprivation before the test (Fig. 11*A*). The results are presented as a preference index of the odorized water for each group of animals (*n* = 13 mice/group), which was calculated as the amount of the odorized water consumed divided by the total amount of liquid consumed (both odorized water and nonodorized water) daily. Since the random chance to drink from each bottle is 50%, the highest concentration of iVA is interpreted as the animal's detection threshold once its preferential index is not statistically significant from 0.5. As shown in Figure 11*B*, the iVA preferential indexes for three groups of control mice reached 50% after iVA concentration was decreased to 10^−6^ or 10^−7^ M, indicating their detection threshold is ∼10^−6^ M. However, the iVA preferential index of the DREADD/CNO group of mice in the concentration range from 10^−5^ M to 10^−3^ M was significantly different from that in each of the other three groups (one-way ANOVA analysis, Fig. 11*B*), suggesting that chemogenetic inhibition of the CCKergic STCs significantly elevates the animal's detection threshold for iVA compared to control mice. Taken together, these findings strongly support the important roles of CCKergic STCs in amplifying odorant signals to MCs thus enhancing the system's sensitivity to odorants.

## Discussion

Five major findings were generated from the present study focusing on how functional modulation of CCKergic STCs, the predominant structural elements of the IAS, with optogenetic and chemogenetic approaches impacts the physiological output of MCs in the OB and the odor-based behavioral outcomes. First, both AMPA and NMDA receptors contribute to the ON-evoked monosynaptic responses in the CCKergic STCs. NMDA receptors mediate the long-lasting and late component of these events. Second, CCKergic STCs provide long-lasting synaptic excitation to MCs via dendrodendritic transmission in the glomerulus. This STC→MC transmission is mediated by both glutamate-mediated chemical and gap junction-based electrical synapses. Third, excitatory STC input significantly amplifies MC output in response to ON stimulation. Fourth, axons from the CCKergic STCs on the opposite side of the same OB provide excitatory feedforward to local MCs. Lastly, functional inhibition of CCKergic STCs impairs odor detection by drastic reduction of animal's sensitivity to odors.

**Figure 12.**
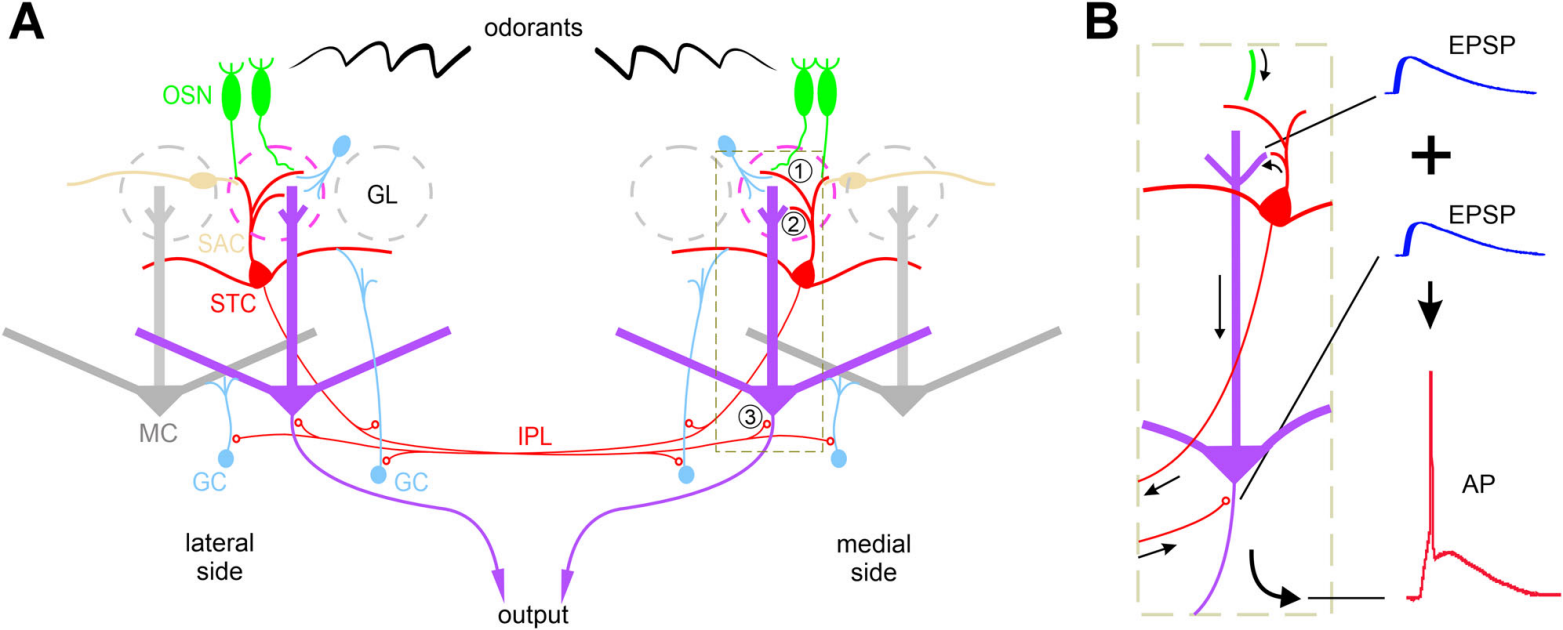
Speculative summary of CCKergic TC actions on MC output. ***A***, STCs receive direct excitatory input from OSNs (1) and intermediate the excitatory input to MCs via apical dendrites (2) and provide excitatory feedforward to MCs on the opposite side of the same OB via their axon terminals in the IPL (3). ***B***, Illustration of summation of the subthreshold orthodromic synaptic response (EPSP) evoked apical dendrites of STCs on the same side and subthreshold antidromic synaptic response (EPSP) elicited by axons of STCs on the opposite side to trigger a suprathreshold output response (action potential, AP) in the MC.

Based on ultrastructural evidence of synaptic connections between CCKerigic TC axons and GC dendrites in the IPL, the IAS was initially proposed to function as a neural circuit linking the infraglomerular inhibitory circuits on the medial and lateral sides of the same bulb (Liu and Shipley, 1994). The functional operation of this axodendritic STC→GC synapse was recently characterized (Sun et al., 2020). Moreover, CCKergic STCs drive two subpopulations of inhibitory glomerular interneurons via their apical dendrites. Intriguingly, among these inhibitory glomerular interneurons and GCs, only the SACs can be activated by CCK at a physiological concentration (Liu and Liu, 2018), suggesting the SAC-mediated lateral inhibition in the OB can be further elevated by endogenous CCK potentially released from CCKergic STCs when they fire high-frequency action potentials in response to repetitive OSN input (Ghijsen et al., 2001; Jones et al., 2020; Sun et al., 2020). All these experimental findings suggest that CCKergic STCs form the IAS and recruit different inhibitory circuits to process odor information in the OB (Fig. 12*A*).

In contrast to their dynamic actions on inhibitory circuits, the present study for the first time provided multiple lines of evidence supporting that CCKergic STCs play a functionally amplifying role in transforming the OB input to output and enabling the system to detect weak odorant stimuli from the environment via their synaptic connections with MCs at multiple levels (Fig. 12*A*).

First, activation of NMDA receptors significantly augments STC output in response to monosynaptic OSN input. This finding aligns with previous studies showing that STCs respond to OSN input in OB slices or odorants especially at low concentration in whole animals with more reliable responses of larger amplitude and shorter latencies or more spikes compared with MCs (Griff et al., 2008; Short and Wachowiak, 2019; Eiting and Wachowiak, 2020; Jones et al., 2020). Although the relatively high input resistance and small soma sizes potentially contribute to the strong CCKergic STC response to OSN input (Jones et al., 2020), the present study provides the pharmacological evidence supporting the contribution of NMDA receptors to enabling the CCKergic STCs to respond to weak odor stimuli.

Second, CCKergic STCs excite MCs in the glomerulus via dendrodendritic chemical transmission and electrical synapses. Optogenetic activation of CCKergic STC apical dendrites elicited a long-lasting inward current or a long train of action potentials in MCs with apical dendrites affiliated with the activated glomerulus. Since CCKergic STCs exclusively receive monosynaptic OSN input (Jones et al., 2020; Sun et al., 2020), this finding implies the contribution of CCKergic STCs to the ON-evoked LLD in MCs (Carlson et al., 2000) like the external tufted cells (eTCs; De Saint Jan et al., 2009; Najac et al., 2011; Gire et al., 2012), a distinct subpopulation of TCs with somata confined to the glomerular layer but lacking lateral dendrites (Macrides and Schneider, 1982; Hayar et al., 2004; Antal et al., 2006). This conclusion is further supported by the observation that the ON-evoked MC responses were significantly reduced by optogenetic inhibition of CCKergic STCs. The STC→MC dendrodendritic transmission qualitatively aligns with a previous study (Zhou and Belluscio, 2008). Dendrodendritic synapses between excitatory neurons in the glomerulus were identified or indicated in previous ultrastructural studies (Hinds, 1970; Kosaka and Kosaka, 2005; Bourne and Schoppa, 2017). However, due to their rarity, we cannot rule out the possibility that the STC→MC responses observed here could be mainly mediated by glutamate spillover, despite their short latencies, which may result from strong optogenetic stimulation of the presynaptic neurons (Gire et al., 2019). Nevertheless, NMDA receptors on MCs play a crucial role in enhancing MC responses to CCKergic STC input via dendrodendritic transmission within the glomerulus.

In addition to the glutamate-mediated transmission, gap junction contributes to a nonnegligible portion (∼23% of initial EPSC amplitude) of the STC-evoked excitatory response in MCs. This observation is consistent with previous work showing gap junctions between intraglomerular mTC dendrites (Kosaka and Kosaka, 2004) and similar to that observed in the eTC–MC connection (Gire et al., 2012). In addition to boosting the glutamate-mediated transmission from STCs to MCs, this electrical synapse could also play a role in synchronizing activities between STCs and MCs in the glomerulus and/or produce a shunting effect between them but to a lesser extent compared with MC–MC gap junction (Christie et al., 2005).

Third, STCs activate MCs on the opposite side of the same bulb via their axons traveling within the IPL. Different from the dendrodendritic transmission between the apical dendrites of CCKergic STCs and MCs in the glomerulus, the glutamate-mediated MC response to optogenetic activation of axons in the IPL projecting from CCKergic STCs on the opposite side of the OB was mainly mediated by AMPA receptors but showed a relatively long onset latency (∼5 ms). Considering the existence of many experimental variables such as the level of ChR2 expression on axon terminals of CCKergic STCs and the depth of the activated axons from the slice surface leading to temporally differential levels of glutamate release, the relatively long onset latency of MC response in this context does not exclude the possible monosynaptic nature of this transmission. Furthermore, there were two cells (one with an intact apical dendrite and the other with a truncated apical dendrite) showed short latencies (2.6 ± 0.1 ms) and small jitter (410.5 µs; Fig. 8*F*), consistent with monosynaptic transmission. Intriguingly, a previous study reported similar response latencies in MCs when the electrical stimulation was given to the IPL on the opposite side under the mirror glomerulus to antidromically activate the IAS-forming TCs with apical dendrites ramifying in the same glomerulus of the recorded MC in a hemibulb slice preparation (Zhou and Belluscio, 2008). In that scenario, the transmission should be monosynaptic between the apical dendrites of TCs and MCs. Additionally, the lack of excitatory interneurons in the IPL does not support polysynaptic transmission from CCKergic TC axons to MCs. Whether this is a spillover action of glutamate released from CCKeric axon terminals in the IPL is worth future work.

The functional significance of STC–MC connections in the IPL on the opposite side of the same OB can be speculated to involve synchronizing MC output from mirror glomeruli based on previous evidence showing that IAS axons terminate precisely beneath mirror glomeruli on the opposite OB side (Belluscio et al., 2002; Lodovichi et al., 2003; Zhou and Belluscio, 2008). However, our study does not provide direct evidence for this hypothesis. Additionally, other possibilities cannot be ruled out, such as the activation of MCs associated with neighboring glomeruli, particularly given the potential branching of STC axons in the IPL (Orona et al., 1984). Alternatively, STC–MC excitation could be counterbalanced by inhibition from local granule cells, which also receive STC axonal input in the IPL (Liu and Shipley, 1994; Sun et al., 2020). This inhibitory influence may be further modulated by strong excitatory input from cortical centrifugal projections, such as those from the piriform cortex (Boyd et al., 2012) or the anterior olfactory nucleus (AON; Markopoulos et al., 2012). Therefore, whether the net functional impact of this circuit on MC output from mirror or relevant glomeruli is excitatory or inhibitory depends on the interplay between MC-derived excitation and GC-mediated inhibition.

Finally, animal's sensitivity to food odor and iVA was significantly reduced by chemogenetic inhibition of the CCKergic STCs. STC axons projecting out of the OB to target the anterior piriform cortex, the par externa of the AON, and the cap part of the olfactory tubercle (Igarashi et al., 2012; Hirata, 2024) could contribute to these effects because these downstream structures may play a role in olfactory perception, odor detection or discrimination, but a straightforward speculation based on findings of the present study is that CCKergic STCs regulate the system's sensitivity to odor stimuli by acting on MCs affiliated with mirror glomeruli via the IAS (Fig. 12*B*).

Taken together, the present study demonstrated glutamatergic excitation of MCs on the medial and lateral sides of the same bulb by the CCKergic STCs. This excitatory feedforward connection could be particularly important for the system to detect weak odor stimuli as we speculate that the simultaneous excitation from the STCs affiliated with mirror glomeruli on both sides of the OB will summate to boost the weak stimulation-produced subthreshold MC responses to the suprathreshold level leading to spike output to downstream centers (Fig. 12*B*). Given that temporal integration of convergent OB output from multiple glomeruli determines the firing probability of pyramidal neurons in the piriform cortex (Zhou and Belluscio, 2008; Apicella et al., 2010), we further speculate that CCKergic STCs function to ensure weak odor detection and representation in the cortex by amplifying and synchronizing MC output from mirror glomeruli. These speculations certainly necessitate future research endeavors.
